# Phylogenomic branch length estimation using quartets

**DOI:** 10.1093/bioinformatics/btad221

**Published:** 2023-06-30

**Authors:** Yasamin Tabatabaee, Chao Zhang, Tandy Warnow, Siavash Mirarab

**Affiliations:** Department of Computer Science, University of Illinois at Urbana-Champaign, Urbana, IL 61801, United States; Department of Integrative Biology, University of California at Berkeley, Berkeley, CA 94720, United States; Department of Computer Science, University of Illinois at Urbana-Champaign, Urbana, IL 61801, United States; Department of Electrical and Computer Engineering, University of California, San Diego, La Jolla, CA 92093, United States

## Abstract

**Motivation:**

Branch lengths and topology of a species tree are essential in most downstream analyses, including estimation of diversification dates, characterization of selection, understanding adaptation, and comparative genomics. Modern phylogenomic analyses often use methods that account for the heterogeneity of evolutionary histories across the genome due to processes such as incomplete lineage sorting. However, these methods typically do not generate branch lengths in units that are usable by downstream applications, forcing phylogenomic analyses to resort to alternative shortcuts such as estimating branch lengths by concatenating gene alignments into a supermatrix. Yet, concatenation and other available approaches for estimating branch lengths fail to address heterogeneity across the genome.

**Results:**

In this article, we derive expected values of gene tree branch lengths in substitution units under an extension of the multispecies coalescent (MSC) model that allows substitutions with varying rates across the species tree. We present CASTLES, a new technique for estimating branch lengths on the species tree from estimated gene trees that uses these expected values, and our study shows that CASTLES improves on the most accurate prior methods with respect to both speed and accuracy.

**Availability and implementation:**

CASTLES is available at https://github.com/ytabatabaee/CASTLES.

## 1 Introduction

Species trees, both their topologies and their branch lengths, are necessary for downstream biological research. For example, branch lengths are required for comparative genomics (Hahn et al. 2005) and comparative trait analysis (Felsenstein 1985; O’Meara 2012), phylodynamics of disease transmission (Volz et al. 2013), species delimitation (Rannala 2015), measuring phylogenetic diversity (Faith 2002; Lozupone and Knight 2005), and detecting and characterizing selection (Kosakovsky Pond and Frost 2005). Many of these analyses amount to studying changes in the rate of evolution across the tree (Lanfear et al. 2010). Most statistical methods designed for these applications rely on branch lengths measured in the unit of the expected number of substitutions per site (SU), readily available from tree inference based on sequence data, or unit of time, or both.

The traditional approach to the estimation of species trees and branch lengths has been concatenating gene alignments followed by a tree-building method, such as maximum likelihood (Rokas et al. 2003). It is now understood (Roch and Steel 2015) that this concatenation approach can be positively misleading (i.e. converge to the wrong tree as the number of genes increases) in the face of sufficient gene tree heterogeneity across the genome due to incomplete lineage sorting (ILS), as modeled by the multispecies coalescent (MSC) model (Pamilo and Nei 1988). Alternative approaches for estimating species trees have been developed that are statistically consistent under the MSC (see Kubatko and Knowles 2023). In particular, methods that combine a set of gene trees to infer a species tree (referred to as “summary methods”) are widely used because of their scalability and accuracy (and notably better accuracy than concatenation when ILS is high). Well-known examples of such methods are ASTRAL (Mirarab et al. 2014) and MP-EST (Liu et al. 2010), which are used often to analyze phylogenomic datasets. However, the branch lengths produced by summary methods are in coalescent units (CUs), and these do not directly lead to branch lengths in substitution units. Moreover, branch lengths in coalescent units are inferable only for the internal branches, which further limits their utility.

At the current time, therefore, most coalescent-based analyses estimate species trees and their branch lengths in substitution units (SU) following a two-stage approach, where the first stage computes the tree topology (e.g. using a summary method, such as ASTRAL or MP-EST) and then estimates branch lengths on the tree using a constrained concatenation analysis, such as using a maximum likelihood method to infer branch lengths on a fixed tree topology (e.g. Song et al. 2012). However, one major problem with this approach is that the branch length calculation step ignores gene tree heterogeneity across the genome, leading to criticisms of this approach in the scientific literature. For example, Moody et al. (2022) criticized the findings by Zhu et al. (2019) who postulated a shorter length than previously reported separating archaea and bacteria, arguing that the use of concatenation for branch length estimation can lead to substantial under-estimation in the face of high levels of horizontal gene transfer, where gene trees have widely discordant topologies.

Another approach for SU branch length estimation on species trees is ERaBLE (Binet et al. 2016), which uses the SU branch lengths estimated in a set of gene trees and then solves a weighted least-squared optimization problem to assign SU branch lengths to the species tree. However, as with the standard concatenation approach, ERaBLE does not take heterogeneity in gene tree *topologies* due to ILS into account. When a strict molecular clock holds, then branch length estimation in the species tree becomes feasible. However, it is well understood that strict clock-based methods have poor accuracy for many datasets where mutation rates change across the tree (Bromham and Penny 2003; Kumar 2005). Hence, clock-based approaches do not offer a viable solution.

To summarize, existing methods to compute SU branch lengths that take discordance between gene trees due to ILS into account, without a strict molecular clock, have not yet been developed. And in particular, we currently lack a theoretical basis for inferring SU lengths for species trees that addresses heterogeneity in gene tree topologies due to ILS, as modeled by the MSC. This is a glaring gap that needs to be filled.

The unsatisfactory state-of-the-art leads us to ask: How can we estimate branch lengths on species trees that are accurate, even in the face of high levels of ILS and that does not depend on a strong molecular clock? We specifically seek a method that has a strong theoretical foundation based on the MSC. We also seek to develop a method that is sufficiently fast that it is scalable to large genome-wide datasets with hundreds to thousands of genes and species. Because we seek to develop scalable methods, Bayesian co-estimation of gene trees and species trees (topologies and branch lengths) is infeasible as even the best of these methods are computationally intensive on smaller datasets with about 50 species and 200 genes (Zimmermann et al. 2014; Ogilvie et al. 2017).

Here, we propose the Coalescent-Aware Species Tree Length Estimation in Substitution-unit (CASTLES) method. The input to CASTLES is a rooted species tree *topology* and a set of inferred gene trees with SU branch lengths, which can have missing data, polytomies, and multiple individuals per species. The output is the species tree furnished with SU lengths on all branches; at the root, only the sum of the lengths of the two root-incident branches is inferred. CASTLES addresses gene tree heterogeneity under the MSC and naturally occurring variation in mutation rates; thus, it does not assume strict molecular clock. Similar to methods like ASTRAL for species tree topology inference, we use a quartet-based approach. We first derive the expected branch length of gene trees that do or do not match a quartet species tree under our model as a function of their CU length and mutation rates. These derivations suggest an algorithm for estimating SU branch lengths, but the approach is cumbersome to implement. Through approximations and simplifications, we derive a much simpler estimator that still retains non-ultrametricity. Going beyond trees with four species in a naive way, iterating over all $\left(\begin{array}{c} n\\ 4 \end{array}\right)$ quartets, would lead to the loss of scalability. Instead, we design a sophisticated dynamic programming algorithm to compute quantities needed by our algorithm in quadratic time. We compare CASTLES to leading alternatives using a simulation study and demonstrate its superior accuracy and speed. Finally, we apply it to a biological dataset.

## 2 Materials and methods

We first describe a model that generates gene trees with SU branch lengths. We then derive the expected gene tree branch lengths under this model for a single quartet. The resulting set of non-linear equations can be (approximately) solved using numerical methods, and will yield values for the model parameters given quantities that can be measured from the gene trees. However, solving these equations is computationally intensive, involves numerical instabilities, may not produce optimal solutions and is cumbersome; therefore, here we present simplifications that give analytical formulas for every branch of a quartet tree. Using equations for the simplified model, we then develop an algorithm that can handle a tree with arbitrary size *n*, including a scalable (quadratic) dynamic programming algorithm to compute averages of quartet branch lengths across gene trees for $\Theta (n^{4})$ quartets. We relegate most proofs to the Supplementary Appendix.

### 2.1 MSC + Substitution model

**Figure 1.**
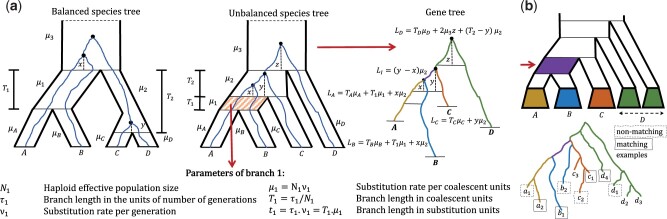
(a) MSC + Substitution model. Each branch of the species tree is furnished with parameters described in the legend. As a gene tree evolves inside the species tree, its branches inherit the substitution rates of all the species tree branches that they pass through. When mutation rates change across species tree branches, the resulting gene tree is non-ultrametric. We match the theoretical expected values of the five branches of a gene tree that matches or does not match the species tree (namely, $L_{A},L_{B},L_{C},L_{D}$, and $L_{I}$ for a matching gene tree shown here) to their empirical means, computed from gene trees. (b) Handling a tree with more than four taxa. Each focal internal branch (arrow) divides the tree into four groups, here denoted as *A*, *B*, *C*, and *D*. To use quartet-based equations, we average branch lengths over all quartets with one leaf selected from each of *A*, *B*, *C*, and *D* (e.g. $a_{1},b_{1},c_{1},d_{1}$). Note that in one gene tree, some quartets around a species tree branch may contribute to matching while others contribute to non-matching average lengths (examples shown). We compute these averages efficiently without listing all $O(n^{4})$ quartets using dynamic programming.

Our model parameters include a species tree T and several per-branch attributes (Fig. 1a). Each branch *i* is furnished with a branch length $\tau_{i}$ in the unit of the number of generations, a haploid effective population size $N_{i}$, and a substitutions-per-generation rate $\nu_{i}$. The CU length of the branch is simply $T_{i}=\tau_{i}/N_{i}$. Let $\mu_{i}=\nu_{i}\times N_{i}$ denote the CU substitution rate. The SU length of the branch is $t_{i}=\tau_{i}\times \nu_{i}=T_{i}\times \mu_{i}$; thus, setting unequal ν values across the tree branches leads to a non-ultrametric species tree. Gene trees are first drawn under the MSC model (ignoring $\nu_{i}$), thus producing trees with lengths in the unit of generations. Gene trees with SU length are generated by multiplying the length of every infinitesimally small part of each of their branches passing through a species tree branch *i* by the species tree rate $\mu_{i}$. For example, the length of the terminal branch *A* in Fig. 1 is $T_{A}\mu_{A}+T_{1}\mu_{1}+x\mu_{2}$. Note that under this model, species tree CU lengths connect indirectly to SU and time units; inferring SU from CU requires $\mu_{i}=\nu_{i}\times N_{i}$, inferring the number of generations needs the population size, and inferring time additionally needs the generation time.

### 2.2 Expected quartet branch lengths under the MSC

Focusing on a quartet, we now derive the expected length of all branches as a function of the model parameters. Consider unbalanced and balanced species trees shown in Fig. 1. In the Supplementary Appendix, we present Supplementary Lemmas S1–S6, which derive the expected length of each terminal and internal branch in the gene trees that do or do not match the unbalanced or balanced species trees. Note that these expectations can be estimated in a statistically consistent manner given true gene trees with SU branch lengths. Combined, we derive 10 equations across the five branches, relating the measurable expected values to the unknown parameters. Since $\mu_{i}$ and $T_{i}$ only appear as $t_{i}=\mu_{i}T_{i}$ for all terminal branches (i∈{A,B,C,D}), we have four unknown parameters for terminal branches. With three unknown rates ($\mu_{1},\mu_{2},\mu_{3}$) and two unknown CU internal lengths ($T_{1}$ and $T_{2}$), we have 9 unknowns in total.

This non-linear system of 10 equations and 9 unknowns can be (approximately) solved using numerical methods to jointly estimate all the parameters. Such an optimization approach, however, is subject to numerical instability, may be slow, and may not give optimal solutions for this (possibly) non-convex optimization problem. Instead of exploring that path, we observe that by making some simplifying assumptions, we can compute all the branch lengths analytically.

Theorem 1 and the similar Supplementary Theorem S1 (Supplementary Appendix) for balanced trees follow from the lemmas mentioned earlier.

Theorem 1 (Unbalanced). *For the unbalanced species tree of Fig. 1a, let*$\Delta_{I}$*be the difference in the expected internal branch length in substitution units of gene trees with an unrooted topology matching the species tree and those not matching the species tree. Then*,

*Similarly, let* $\Delta_{A},\Delta_{C},$*and* $\Delta_{D}$*be the difference in the expected length of matching and non-matching gene trees for the terminal branch leading to a cherry, the middle terminal branch, and the root-adjacent terminal branch, respectively.*

(1) $$\begin{array}{c} \Delta_{I}=\frac{3(e^{-T_{2}}-e^{-3T_{2}})(1-e^{-T_{1}})(\mu_{2}-\mu_{3})+6\mu_{1}(e^{-T_{1}}-1+T_{1})}{2(3-2e^{-T_{1}})} \end{array}.$$

(2) $$\begin{array}{c} \Delta_{A}=\frac{4\mu_{2}-6\mu_{1}-(3e^{-T_{2}}+e^{-3T_{2}}+\frac{9}{2}e^{T_{1}-T_{2}})(\mu_{2}-\mu_{3})}{2(-2+3e^{T_{1}})}\\ +\frac{e^{T_{1}}\left(\frac{1}{2}(\mu_{2}+\mu_{3})e^{-3T_{2}}+6\mu_{1}(1-T_{1})-5\mu_{2}\right)}{2(-2+3e^{T_{1}})} \end{array}.$$

(3) $$\begin{array}{c} \Delta_{C}=\frac{(2-e^{-T_{1}})\left(\left(e^{-3T_{2}}+2\right)\mu_{2}-3e^{-T_{2}}(\mu_{2}-\mu_{3})\right)}{2(3-2e^{-T_{1}})}\\ +\frac{\mu_{3}e^{-3T_{2}}(e^{-T_{1}}-4)}{2(3-2e^{-T_{1}})} \end{array}.$$

(4) $$\begin{array}{cc} \Delta_{D}= & \frac{\left(1-e^{-T_{1}})(2\mu_{2}-(3e^{-T_{2}}-e^{-3T_{2}})(\mu_{2}-\mu_{3})\right)}{2(3-2e^{-T_{1}})} \end{array}.$$

We simplify the equations of Theorem 1 by computing their limit as $T_{2}\to \infty$ or $\mu_{2}\to \mu_{3}$. Note that neither assumption completely breaks non-ultrametricity assumptions because we ignore the rate for only one branch ($\mu_{2}$) and not the others.

### 2.3 Simplifications

#### 2.3.1 Internal branch calculation

To compute $t_{1}=\mu_{1}T_{1}$, we simplify Equation (1) so that it only depends on $T_{1}$ and $\mu_{1}$, by computing its limits:


(5) $$\begin{array}{c} \underset{T_{2}\to \infty }{\lim}\Delta_{I}=\underset{T_{2}\to 0}{\lim}\Delta_{I}=\underset{\mu_{2}\to \mu_{3}}{\lim}\Delta_{I}=\frac{3\mu_{1}(e^{-T_{1}}-1+T_{1})}{3-2e^{-T_{1}}} \end{array}.$$

Replacing $\Delta_{I}$ with the observed difference between mean internal branches among matching and non-matching gene trees (${\bar{\Delta }}_{I}$), we get an equation with two unknowns, $\mu_{1}$ and $T_{1}$. One way to move forward is to estimate $T_{1}$ using quartet discordance, as shown by Sayyari and Mirarab (2016). Then, we can estimate $\mu_{1}$ and thus $t_{1}=\mu_{1}\times T_{1}$. However, the accuracy of the CU estimate of $T_{1}$ is known to degrade for inaccurate gene trees (Sayyari and Mirarab 2016). Instead, we use a local clock approximation to estimate $\mu_{1}$ and then solve for $T_{1}$. If mutation rates of the two branches above the focal branch are assumed the same (e.g. $\mu_{2}=\mu_{3}$), then, the expected length of gene trees not matching the species tree is simply $\mu_{2}$ by Supplementary Lemma S1 (Supplementary Appendix). Further assuming $\mu_{1}=\mu_{2}$ allows us to estimate $\mu_{1}$ as the mean length of the internal quartet branch among gene trees not matching the species tree ($\mu_{1}={\bar{L}}_{I}^{\prime }$), obtaining:


(6) $$\begin{array}{cc} \frac{{\bar{\Delta }}_{I}}{{\bar{L}}_{I}^{\prime }} & =\frac{3(T_{1}+e^{-T_{1}}-1)}{3-2e^{-T_{1}}} \end{array}.$$

The solution to this equation is: where W(.) is the Lambert W function and $\bar{\delta }={\bar{\Delta }}_{I}/{\bar{L}}_{I}^{\prime }$. Since Lambert’s function does not have a closed-form solution (and can be imaginary), we resort to the Taylor expansion $e^{T_{1}}\approx 1+T_{1}$, which is a good approximation for small $T_{1}$. Using this approximation, the solution to Equation (6) becomes:


$$\bar{\delta }+W\left(-\frac{1}{3}e^{-\bar{\delta }-1}(2\bar{\delta }+3)\right)+1,$$

(7) $$\hat{T_{1}}=\frac{1}{2}\bar{\delta }+\frac{1}{6}\sqrt{3\bar{\delta }(3\bar{\delta }+4)}.$$

In our current implementation of CASTLES, we use this approximation to avoid numerical issues. When δ<0, we set the branch length to a small value ($10^{-6}$ by default).

#### 2.3.2 Terminal branch calculation

To simplify Equation (2), we compute its limit as $T_{2}\to \infty$


(8) $$\begin{array}{cc} \underset{T_{2}\to \infty }{\lim}\Delta_{A} & =\frac{-6\mu_{1}(e^{-T_{1}}-1+T_{1})-(5-4e^{-T_{1}})\mu_{2}}{6-4e^{-T_{1}}}\;. \end{array}$$

The expected length of the terminal branch of *A* in non-matching gene trees in the limit is based on Supplementary Equation (S19) (Supplementary Appendix). To compute $t_{A}=\mu_{A}T_{A}$ we replace the expected value $\underset{T_{2}\to \infty }{\lim}\Delta_{A}$ in Equation (8) with the observed mean difference ${\bar{\Delta }}_{A}$ and replace the expected value $\underset{T_{2}\to \infty }{\lim}L_{A}^{\prime }$ in Equation (9) with the observed mean terminal branch of *A* among non-matching gene trees (${\bar{L}}_{A}^{\prime }$). Solving for $T_{A}\mu_{A}$ gives us an estimator of $t_{A}$:


(9) $$\underset{T_{2}\to \infty }{\lim}L_{A}^{\prime }=T_{1}\mu_{1}+T_{A}\mu_{A}+\frac{5}{6}\mu_{2}$$

(10) $$\hat{t_{A}}={\bar{L}}_{A}^{\prime }+\frac{\mu_{1}(e^{-T_{1}}-1+T_{1})+{\bar{\Delta }}_{A}(1-2/3e^{-T_{1}})}{1-4/5e^{-T_{1}}}-T_{1}\mu_{1}\;.$$

Similarly, for branch *C*, where the expected length of *C* in non-matching gene trees ($L_{C}^{\prime }$) is given in Supplementary Equation (S6) in the Supplementary Appendix. Replacing $\underset{T_{2}\to \infty }{\lim}L_{C}^{\prime }$ with the observed length of *C* in non-matching gene trees ${\bar{L}}_{C}^{\prime }$ and replacing $\underset{T_{2}\to \infty }{\lim}\Delta_{C}$ with the observed ${\bar{\Delta }}_{C}$ in Equation (11) gives us the estimate for $t_{C}=T_{C}\mu_{C}$:


(11) $$\underset{T_{2}\to \infty }{\lim}\Delta_{C}=\frac{\mu_{2}\left(2-e^{-T_{1}}\right)}{\left(3-2e^{-T_{1}}\right)}\;\text{and}\;\underset{T_{2}\to \infty }{\lim}L_{C}^{\prime }=\frac{1}{3}\mu_{2}+T_{C}\mu_{C},$$

(12) $$\hat{t_{C}}={\bar{L}}_{C}^{\prime }-\frac{1}{3}(2-\frac{1}{2-e^{-T_{1}}}){\bar{\Delta }}_{C}\;.$$

For *D*, we use a different limit: where the expected length of *D* in non-matching gene trees ($L_{D}^{\prime }$) is given in Supplementary Equation (S17) in the Supplementary Appendix. The pendant branch of *D* in SU in the *unrooted* species tree is $\mu_{2}T_{2}+\mu_{D}T_{D}$, representing both branches below the root. Substituting expected values $\Delta_{D}$ and $L_{D}^{\prime }$ with observed values ${\bar{\Delta }}_{D}$ and ${\bar{L}}_{D}^{\prime }$, we get our estimate:


$$\begin{array}{c} \underset{\mu_{2}\to \mu_{3}}{\lim}\Delta_{D}=\frac{\mu_{2}\left(1-e^{-T_{1}}\right)}{3-2e^{-T_{1}}}\\ \underset{\mu_{2}\to \mu_{3}}{\lim}L_{D}^{\prime }=\mu_{2}T_{2}+\mu_{D}T_{D}+\frac{2}{3}\mu_{2} \end{array},$$

(13) $$\hat{t_{2}}+\hat{t_{D}}={\bar{L}}_{D}^{\prime }-\frac{2}{3}(2+\frac{1}{1-e^{-T_{1}}}){\bar{\Delta }}_{D}\;.$$

To summarize, we use Equations (10), (12), and (13) to compute terminal branch lengths, setting the length to a small value ($10^{-6}$ by default) when results are negative.

### 2.4 Extending to larger trees

To extend the algorithm to more than four species, we apply the same calculations to each branch of the species tree, one at a time. Each internal branch of the species tree creates a quadripartition of species (e.g. A,B|C,D in Fig. 1b). Any quartet of species (e.g. ab|cd) with a selection of one taxon from each part of the quadripartition (a∈A, b∈B, c∈C, and d∈D) gives us a quartet species tree where all of our previous theoretical results hold, and they all lead to identical expected values for their corresponding gene tree quartets. Thus, it is valid to compute the length of this species tree branch using the quartet-based approach by simply taking the average lengths across all quartets.

Assuming the averages are already calculated, we can use Algorithm 1 to assign a length to each branch. The algorithm visits the internal nodes of the tree in a post-order traversal. For each internal node, it assigns the length of the edge above, in addition to (some of the) adjacent terminal branches. If a node *u* is the parent of a cherry, it assigns the length to both children; otherwise, it ignores the children. If *u* is sister to a leaf, it also assigns the length to the sister, using Equation (12). When the tree has more than four taxa, almost all branch lengths are assigned using unbalanced quartet equations. The only exception is the root branch, which may need to be set based on the balanced quartet equations (Supplementary Fig. S5).

The most challenging part of this algorithm, then, is computing mean length across $O(n^{4})$ quartets in a scalable fashion. These quantities can be computed using a sophisticated dynamic programming algorithm, which borrows many ideas from weighted ASTRAL (Zhang and Mirarab 2022). The running time of the algorithm is $O(n^{2}k)$ for *n* leaves and *k* genes. Due to space limitations, we present the full details for this algorithm in the Supplementary Appendix, Section S2.

Algorithm 1. CASTLES algorithm. The input is a rooted species tree *s* with n>4 taxa and a set of gene trees G with SU branch lengths, and the output is *s* annotated with SU branch lengths. $t_{e}$ denotes the length of branch *e* in SU.

1: **procedure** CASTLES(*s*, G)

2:  ${\bar{L}}_{a},{\bar{L}}_{b},{\bar{L}}_{v},{\bar{L}}_{p},{\bar{L}}_{a}^{\prime },{\bar{L}}_{b}^{\prime },{\bar{L}}_{v}^{\prime },{\bar{L}}_{p}^{\prime }$ for each branch ←Supplementary Algorithm S1

3:  **for**u∈ post order traverse of internal nodes of *s* **do**

4:   **if** *u* is root **then**

5:    break

6:   **end if**

7:   p←parent(u);v←sibling(u); a,b←children(u)

8:   **if** *p* is root **then**

9:    **if** *v* is leaf **then**

10:     $t_{p\to u}+t_{p\to v}\leftarrow \text{Equation}\;(13)\;(\text{terminal}\;D)$

11:    **else**

12:     $t_{p\to u}+t_{p\to v}\leftarrow \text{Equation}\;\;(S39)\;(\text{internal bal}.)$

13:    **end if**

14:   **else**

15:    $t_{p\to u}\leftarrow \text{Equation}\;(7)\;(\text{internal unbal}.)$

16:    **if** *v* is leaf **then**

17:     $t_{p\to v}\leftarrow \text{Equation}\;(12)\;(\text{terminal}\;C)$

18:    **end if**

19:    **for**w∈children(u)**do**

20:     **if** *w* is leaf **and**$t_{u\to w}$ is **null then**

21:      $t_{u\to w}\leftarrow \text{Equation}\;(10)\;(\text{terminal}\;A)$

22:     **end if**

23:    **end for**

24:   **end if**

25:  **end for**

26: **end procedure**

### 2.5 Experimental setup

#### 2.5.1 Overview

We performed a simulation study comparing CASTLES to four other methods by estimating branch lengths on the fixed *true* species tree topology. We report error measured as the absolute error averaged over all branches of each tree. Since absolute error hides the contribution of bias versus variance, we also report the mean error (without absolute), which is a valid measure of the bias of a method. Since the mean absolute error emphasizes long branches more than short branches, we also report two metrics that emphasize shorter branches successively more: root mean squared error (RMSE) and mean absolute log error. For all the methods, negative and zero branch lengths are replaced with $10^{-6}$ (the pseudo-count used by RAxML for identical sequences). Since negative lengths are not usable in downstream analyses, this step emulates practice.

We performed two experiments: The first, using a new simulated quartet dataset, is not meant to be realistic but to examine accuracy under idealized conditions and to show the impact of successively more challenging models of rate variation and the level of ILS. The second experiment uses two previously published simulated datasets with larger (30-taxon and 101-taxon) trees and more realistic settings, and examines the effect of gene tree estimation error (GTEE), the level of ILS, rate heterogeneity and deviation from the molecular clock, and the inclusion of an outgroup. Additional information about the simulation are provided in Supplementary Appendix, Section S3.

#### 2.5.2 Datasets

To measure the accuracy, we need a species tree with SU branch lengths. The leading simulation method (SimPhy, Mallo et al. 2016) produces species trees in the unit of the number of generations ($\tau_{i}$). However, SimPhy does select a global substitution rate ν and assigns a mutation rate multiplier ($r_{i}$) to each species tree branch (-hs option); setting $\nu_{i}=r_{i}\times \nu$ matches with the assumed model. Thus, the SU lengths on the species tree can be easily defined as $t_{i}=\tau_{i}\times \nu_{i}$. Unfortunately, SimPhy does not output the $r_{i}$ rates; we modified its code to output these and the species tree with SU lengths. We used this modified version of SimPhy to regenerate species trees used in our datasets mentioned below and confirmed that the same trees are generated. This procedure gives us the ground truth SU lengths. We use SimPhy to evolve gene trees within each model species tree under the MSC, which allows us to explore the impact of ILS on branch length estimation. We quantify the level of ILS using the “Average Distance” (AD) between true species trees and true gene trees, in terms of the normalized Robinson–Foulds (RF) (Robinson and Foulds 1981) distance, producing values that can range from 0% (no discordance) to 100% (no shared branches).

**Quartet dataset.** We generated a new quartet dataset using the modified version of SimPhy. We created six different model conditions by changing the level of ILS (by varying population size) and varying rate heterogeneity multipliers. Our model conditions start from a strict molecular clock with no rate variation (i.e. *Homogeneous*) and becomes successively more complex. Next, we add rate variations across species tree branches only (-hs option), creating a model (*Sp*) akin to MSC + Substitution mentioned earlier. We then create models that have rate variation only across genes but not species (*Loc* using -hl) and both across species and across genes (*Sp, Loc* using -hs -hl). Finally, we add rate variations specific to each branch of each gene tree (*Sp, Loc, Sp/Loc*: -hs -hl -hg), which creates heterotachy; this most complex model is how Simphy is usually used (e.g. in the next datasets) and goes beyond our theoretical model. The first five conditions have an AD = 0.29, indicating a moderate level of ILS. The final condition increases the ILS level to 0.51 AD. Each model condition has 200 replicates, each with 10,000 true gene trees. We intentionally used a large number of true gene trees to verify our formulas and compare methods in an ideal situation. Further details and parameters are provided in Supplementary Tables S5 and S6.

**S100 dataset.** We used a 101-taxon simulated dataset from Zhang et al. (2018) (100 ingroup and one outgroup), that had model conditions characterized by different levels of GTEE, ranging from 0 (for true gene trees) to 0.55, measured in terms of the RF distance between true and estimated gene trees. The ILS level changes dramatically across replicates (average: 0.46 AD). The estimated gene trees were created using FastTree2 (Price et al. 2010). These datasets had 50 replicates, each with 1000 gene trees.

**MVRoot dataset.** We used a 30-taxon dataset from Mai et al. (2017) that had model conditions that varied in terms of deviation from the molecular clock and inclusion of an outgroup. Deviation from the clock was specified with the parameter α of the gamma distribution, choosing 0.15 (*High* variation), 1.5 (*Med*), or 5 (*Low*). This dataset had 100 replicates with 500 gene trees (estimated using FastTree2) in each replicate. The replicates were highly heterogeneous in terms of ILS and GTEE level (average 0.46 AD and 0.38 GTEE across all model conditions).

#### 2.5.3 Methods compared

We compare CASTLES to four other methods: concatenation using maximum likelihood, FastME (Lefort et al. 2015) on two different distance matrices, and ERaBLE (Binet et al. 2016):

- Concatenation with maximum likelihood using RAxML (Stamatakis 2014) is perhaps the dominant method used in the literature, and estimates branch lengths on the given species trees assuming all the sites in the concatenated alignment evolve down a single model tree.
- FastME (Lefort et al. 2015) can estimate branch lengths using the balanced minimum evolution criterion given a distance matrix. We use it with two distance matrices. First, we compute the patristic (path-length) distance between pairs of taxa for each gene tree using Dendropy (Sukumaran and Holder 2010). Genes with no signal (all branch lengths zero) are excluded. We then take either the average or the minimum for each pair across genes. In the absence of rate heterogeneity, the minimum is appropriate and has been used in GLASS and its variants (Mossel and Roch 2010).
- ERaBLE (Binet et al. 2016) is specifically designed for branch-length estimation from a set of gene trees and is similar to FastME but uses weighted means.

## 3 Results

### 3.1 Quartet simulations

**Figure 2.**
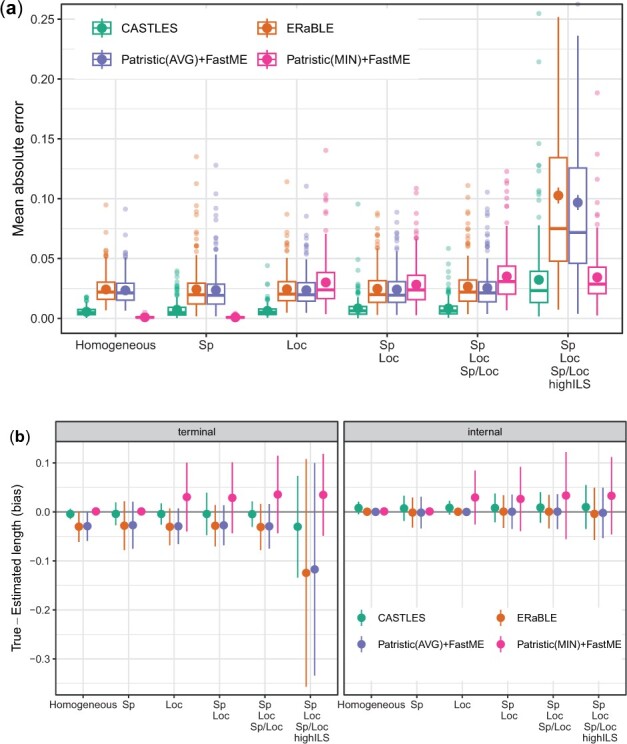
Quartet datasets: mean absolute error (a) and bias (b) of branch lengths estimated using different methods. From left to right, the conditions include more rate variation or higher ILS, creating more challenges for branch length estimation. (a) Mean and standard error across replicates in addition to boxplots. The *y*-axis is cut at 0.25, eliminating 16 outlier cases with unusually high errors (none from CASTLES). (b) Mean and standard deviation.

When considering all conditions, CASTLES has the best accuracy overall (Fig. 2a). Patristic(MIN) + FastME has the lowest error in conditions with no rate heterogeneity across loci. As soon as rate heterogeneity across loci is added (i.e. *Loc*), it goes from being the best method to being the worst. As expected, the error for all methods tends to increase as the models become more challenging (i.e. more rate variation or higher ILS). In the penultimate condition with default ILS and all sources of rate variation, CASTLES has substantially lower error than alternatives. When ILS is increased, we observe a huge increase in error for ERaBLE and Patristic(AVG) + FastME, but not for Patristic(MIN) + FastME. Since the mean absolute error emphasizes long branches more than short branches, we also examine RMSE and log error (Supplementary Fig. S11). The trends with these metrics are very similar to mean absolute error, except that with the log error (emphasizing short branches and long branches alike), Patristic(MIN) + FastME is far worse than the other methods in conditions with rate variation across loci.

Switching from accuracy to bias, we observe little or no bias for CASTLES for terminal branches in all conditions except at the highest ILS level (Fig. 2b and Supplementary Fig. S12). In contrast, ERaBLE and Patristic(AVG) + FastME, have a clear over-estimation bias for terminal branches, and Patristic(MIN) + FastME has a clear underestimation bias (for all branches), except in the absence of rate variation across genes (signified by *Loc*). Terminal branches seem particularly biased in the condition with the highest rate variation and the highest level of ILS. In this condition, while CASTLES does seem to have some bias for terminal branches, it is far less biased than alternative methods. Comparing the last two conditions, we observe that higher ILS has a larger impact on bias than rate variation. In contrast to terminal branches, for internal branches, ERaBLE and Patristic(AVG) + FastME also have low bias (slightly lower than CASTLES).

### 3.2 101-taxon ILS simulations

**Figure 3.**
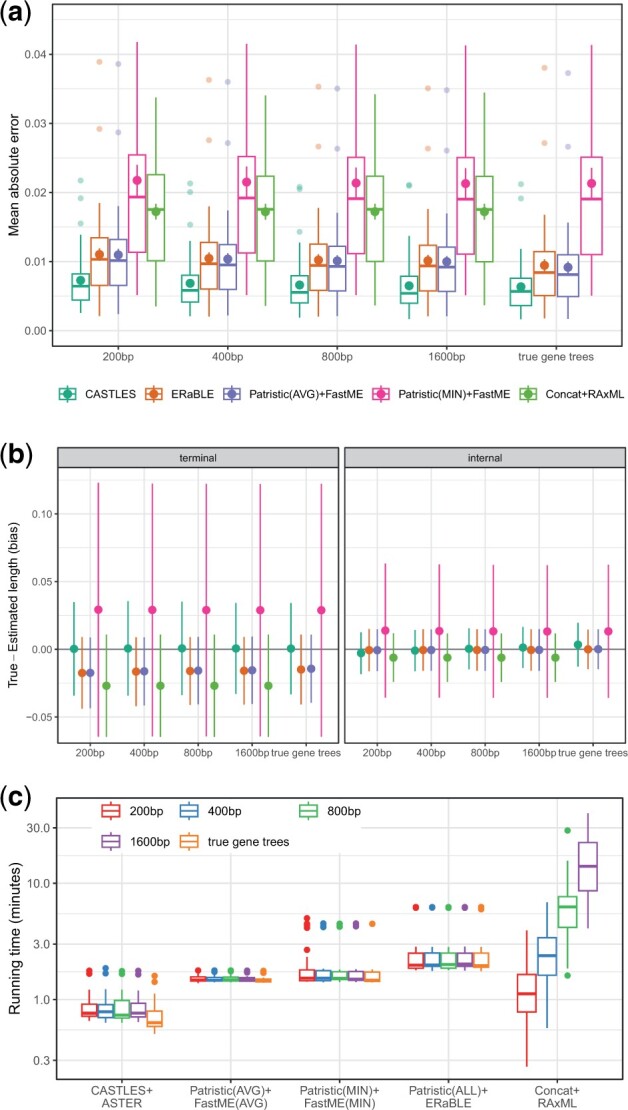
101-taxon datasets: mean and standard error of mean absolute error (a) and mean and standard deviation of bias (b) of branch lengths estimated using different methods. The average GTEE level varies between 0% (for true gene trees) to 23% (for 1600 bp) and then to 55% (for the 200 bp sequences). The number of genes is 1000 and the results are shown across 50 replicates. The *y*-axis is cut at 0.045, eliminating ten outlier cases (none from CASTLES). (c) Running time (log scale), including distance matrix calculation and species tree annotation (by mean branch lengths) but not gene tree estimation; concatenation includes branch length estimation for fixed topology.

On this dataset, CASTLES has the best accuracy across all model conditions, followed by Patristic(AVG) + FastME and ERaBLE, which are very similar to each other (Fig. 3b). Concat + RAxML has substantially higher errors than these three methods that use gene trees as input. However, Patristic(MIN) + FastME has the highest error in all conditions. These patterns remain largely similar, according to the RMSE and log error (Supplementary Fig. S14).

CASTLES shows no substantial bias for terminal branches regardless of the level of gene tree error and a small bias for internal branches (Fig. 3 and Supplementary Fig. S13). This bias is toward under-estimation for true gene trees and gradually moves toward over-estimation as gene tree error increases. In contrast to CASTLES, ERaBLE, Patristic(AVG) + FastME, and Concat + RAxML have a large over-estimation bias for terminal branches. ERaBLE and Patristic(AVG) + FastME have a negligible bias for internal branches. Concat + RAxML has the highest over-estimation bias and is the only method with a substantial over-estimation bias for internal branches. Patristic(MIN) + FastME has a large under-estimation bias. Similar to quartet simulations, all methods are less biased for internal branches than terminal ones. Comparing conditions, we observe that the level of gene tree error has a relatively small impact on under/overestimation for all the methods tested.

On this relatively large dataset, we also examine running times and observe that CASTLES is substantially faster than alternatives (Fig. 3c). Note that gene tree estimation running time is not included for methods based on gene trees because those are often inferred *regardless* of branch length estimation. Concat + RaxML becomes successively slower and uses more memory (Supplementary Fig. S15) as the genes become longer. In the most extreme case, CASTLES can be more than an order of magnitude faster than Concat + RAxML.

### 3.3 30-taxon MVRoot simulations

**Figure 4.**
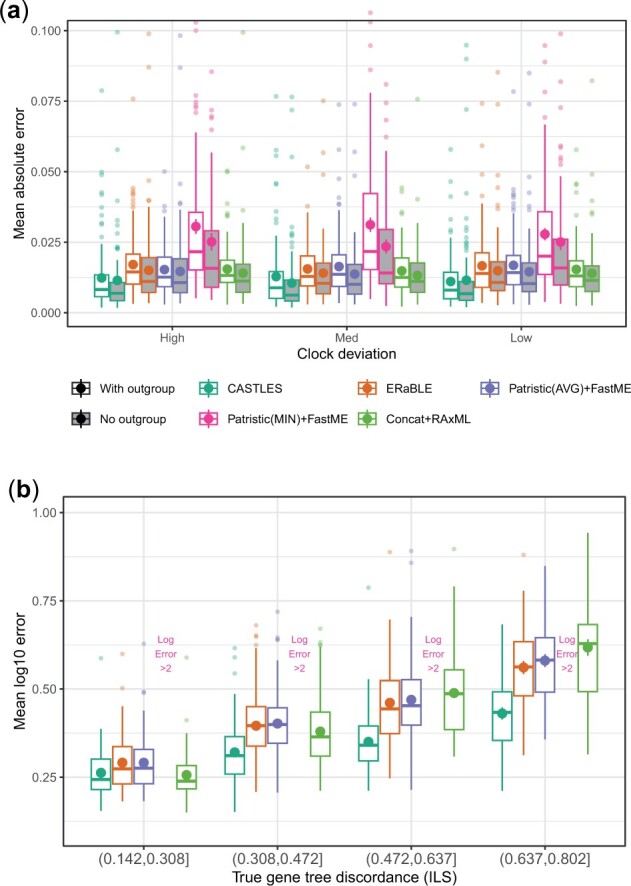
30-taxon MVRoot dataset. (a) Mean absolute error of estimated branch lengths on the 30-taxon MVRoot dataset, with or without an outgroup and with different levels of deviation from a strict clock. The number of genes is 500 and the results are shown across 100 replicates; the *y*-axis is cut at 0.11, leaving 16 outliers out of the graph (one from CASTLES). (b) Focusing on cases without outgroups, we divide replicates based on their level of true gene tree discordance due to ILS into four groups. We show mean log error to control for the correlation between ILS and branch length. Patristic(MIN) + FastME has mean log error above 2 (see Supplementary Fig. S18) and is excluded.

On the 30-taxon MVRoot datasets, we further evaluate the impact of outgroups, deviation from the clock, and ILS level (Fig. 4). Whether an outgroup is included and independent of deviation from the clock, CASTLES has the lowest error (Fig. 4a). On these datasets, CASTLES has no discernible bias, ERaBLE, Patristic(AVG) + FastME and Concat + RAxML have a bias toward over-estimation (see an example replicate in Supplementary Fig. S19a), and Patristic(MIN) + FastME has a more severe bias toward under-estimation; outgroup inclusion and deviation from the clock impact the bias of methods only marginally (Supplementary Fig. S16). For all methods, including an outgroup leads to an increase in the mean absolute error (Fig. 4a). Increasing deviation from the clock does not substantially impact the accuracy of CASTLES or other methods (Fig. 4 and Supplementary Fig. S16).

To compare across levels of ILS, we resort to the logarithmic error because true branch lengths correlate with ILS (i.e. are shorter for higher ILS), and hence, the absolute error confuses the interpretation of the impact of ILS. Across all ILS levels (Fig. 4b), Patristic(MIN) + FastME has very poor performance in terms of log error and is not further discussed below. With the lowest ILS, CASTLES and Concat + RAxML have very similar performance. As ILS increases, all methods become less accurate, but CASTLES degrades in accuracy *slower* than the rest of the methods and hence dominates the other methods for accuracy, with ERaBLE in second place. Concat + RAxML matches CASTLES for the lowest ILS but gradually moves to be the second least accurate of all methods (only better than Patristic(MIN) + FastME) at the highest ILS level. In fact, Concat + RAxML is substantially more sensitive to ILS ($R^{2}=0.57$ Pearson correlation with AD; Supplementary Fig. S17) than CASTLES ($R^{2}=0.23$). Comparing the relative accuracy of methods as ILS changes using the mean absolute error shows similar trends (Supplementary Fig. S18) with one notable difference: Concat + RAxML is *better* than CASTLES at the lowest ILS level but is worse in other conditions (just as with the log error).

### 3.4 Mammalian biological dataset

We apply CASTLES and Concat + RAxML on the 37-taxon mammalian dataset of Song et al. (2012) (perhaps the first paper that used concatenation to estimate branch lengths on a species tree estimated using a summary method) after removing 23 mislabeled gene trees, retaining 424 genes (Supplementary Fig. S19b). We observe patterns similar to the simulated dataset. Branch lengths tend to be longer using Concat + RAxML than using CASTLES (Supplementary Fig. S20). For example, primates are roughly twice as distant from the root of placental mammals in the Concat + RAxML tree as they are in the CASTLES tree. While the two trees are similar in their longest branches and have similar diameters (i.e. from rat to platypus), many of the other internal branches are substantially shorter in CASTLES. While the truth is not known on real data, we note that a similar pattern is observed in simulations, and in simulations, Concat + RAxML is biased toward over-estimation; in contrast, CASTLES is far less biased (e.g. Supplementary Fig. S19a).

## 4 Discussion

Although CASTLES was almost universally more accurate than the competing methods, comparisons across the experiments revealed some interesting trends. Concatenation performed well on low ILS cases and much worse with high ILS, as expected. Increasing ILS did increase error for all methods but also note that more ILS in our simulation is often (though not always) accompanied by shorter branch lengths, which are in general harder to estimate (Supplementary Figs S12 and S13). However, the fact that concatenation degraded in accuracy faster than other methods as ILS increased confirmed that it is less able to deal with gene tree discordance. Thus, the current standard method (concatenation) does suffer from a predictable shortcoming. CASTLES is meant to address that shortcoming.

We observed that estimating terminal branches was harder than internal branches across all datasets for all methods other than CASTLES. As we expected, methods that ignore coalescent (e.g. concatenation and FastME based on *average* patristic distance) had a consistent overestimation bias. What was surprising was that this overestimation showed its effects more on terminal branches than internal branches. The reasons for this clear trend are not clear to us.

Another consistent pattern was that including an outgroup reduced accuracy for all methods and especially for CASTLES. Outgroups are often connected via long branches and have been found problematic for phylogenetic inference and downstream analyses (Li et al. 2012). Our results suggest that they can also confound SU branch length estimation. While not surprising, this pattern suggests that unless the outgroup is needed for a downstream analysis, the outgroups should be removed after rooting the species tree and before estimating branch length.

We surprisingly saw little impact caused by GTEE and deviation from a clock. The robustness to deviation from the strict clock can perhaps be explained by the fact that none of the methods used here other than Patristic(MIN) + FastME assume a clock. Note that in CASTLES, even with our simplifying assumptions, each branch is at the end assigned a different mutation rate (the calculation of which assumes surrounding branches have the same rate). The lack of sensitivity to per-gene signal (controlled here by sequence length) is more surprising, especially for the coalescent-based CASTLES. One possibility is that while short sequences can affect the estimated gene tree topologies, they have a more subdued effect on distances within gene trees (Moshiri and Mirarab 2018); thus, even given short sequences, estimated branch lengths (which change in a continuous space) are broadly consistent with true values, especially when averaged over genes. In contrast, CU branch lengths are sensitive to gene tree error, but these are not used in CASTLES.

Our theory did not explicitly discuss rate heterogeneity across genes. However, across-gene rate changes do not impact our calculations under reasonable models of rate variation. Assume that each gene tree is scaled up or down by a constant factor drawn i.i.d. from some rate multiplier distribution with expected value one and independently from the MSC process. Under any such model, all the derived expected values remain intact and hence the method remains valid. It is easy to see the same is not true for GLASS-like distances that involve taking the minimum across genes: they only work if all the genes have equal rates. When rates of evolution of genes are allowed to vary, as the number of genes goes to infinity, all estimated branch lengths go to zero; a pattern that would imply under-estimation bias, as we observed in our data. More broadly, if rate changes are not i.i.d and correlate with other factors such as missing data, accounting for them becomes far more difficult.

Finally, we note that the estimation of terminal branches in CASTLES is sensitive to rooting of the species tree; hence, care must be applied in rooting the species tree before running CASTLES. When an outgroup is not available, the species tree root is identifiable under the MSC and can be inferred using QR-STAR (Tabatabaee et al. 2022a,b) in a statistically consistent manner. Alternative methods such as tripVote (Mai and Mirarab 2022) or methods that assume a strict molecular clock (e.g. midpoint rooting) are also available, though they do not enjoy the same theoretical guarantees.

## 5 Conclusion

We proposed CASTLES, a method for estimating branch lengths of a given species tree using gene tree branch lengths. CASTLES uses derivations made under the MSC model to design a set of coalescent-based equations that correct for the fact that under the MSC, gene trees can be substantially longer than the species tree. Our study provided evidence that CASTLES produces highly accurate branch lengths in substitution units (SUs), improving on prior methods under a wide range of model conditions.

There are several directions for future work. For example, the derivation of CASTLES assumed that the input gene trees differed from the species tree due to ILS alone. This work could be extended to the case where genes evolve within the species tree due to gene duplication and loss as well as ILS. Developing methods for branch length estimation in that context could be potentially enabled through the DISCO (Willson et al. 2022) technique, which replaces every gene family tree by a set of single-copy gene trees, which could then be passed to CASTLES for branch length estimation on the given species tree. A related question left for future work is whether CASTLES is robust to the presence of horizontal transfer or gene flow. Finally, the behavior of the method should be tested when inputs have low taxon occupancy across genes.

Another question of interest is whether CASTLES is a statistically consistent estimator of SU branch lengths. Given that CASTLES is coalescent-based and that we use expected values under the model, we consider this likely, but two technical challenges need to be addressed. First, for the method to be consistent, we need the model to be identifiable, and we did not establish identifiability in this article. Thus, we ask: Is it possible to design different sets of mutation rates and CU lengths that lead to the same patterns of gene tree distribution? If not, are the expected values enough to uniquely identify branch lengths or are higher moments necessary? Moreover, while we had a system of equations that could be optimized directly, we opted for a more stable approach that had several simplifications. It is possible (and perhaps likely) that those simplifications could result in inconsistent branch length estimation. These questions will need to be addressed in future work, and may require that we explore a more complex estimation scheme that does not rely on simplifications.

## Supplementary Material

btad221_Supplementary_Data

## Data Availability

The code, datasets, and scripts used in this study are available at https://github.com/ytabatabaee/CASTLES.
