# Supplementary material for: Phylogenomic branch length estimation using quartets
Source: Bioinformatics. 2023 Jun 30;39(Suppl 1):i185–93. doi: 10.1093/bioinformatics/btad221 (PMC10311336; doi:10.1093/bioinformatics/btad221)
Supplement: btad221_Supplementary_Data [file btad221_supplementary_data.pdf]

Supplementary Materials for "Phylogenomic branch length estimation using quartets"

Yasamin Tabatabaee[1], Chao Zhang[2,3], Tandy Warnow[1], Siavash Mirarab[4]

[1] Department of Computer Science, University of Illinois at Urbana-Champaign, Urbana, IL, USA
[2] GLOBE Institute, University of Copenhagen, Copenhagen, Denmark
[3] Department of Integrative Biology, University of California, Berkeley, Berkeley, CA, USA
[4] Department of Electrical and Computer Engineering, University of California San Diego, San Diego, CA, USA

Contents

S1 Proofs for Branch Length Equations **3**
S1.1 Unbalanced species trees (Theorem 1 and related lemmas) 4
S1.2 Balanced Species Tree 12

S2 Dynamic Programming Algorithm **19**

S3 Details of the Experimental Study **24**
S3.1 Quartet Simulations 24
S3.2 Software Commands and Version Numbers 25
S3.2.1 Branch Length Estimation 25
S3.2.2 Error Calculation 26
S3.2.3 Runtime and Memory 27
S3.3 Biological Data Analysis 27

S4 Additional Figures **28**

List of Figures

S1 Scenarios for unrooted gene tree matching the unbalanced species tree. 5
S2 Scenarios for unrooted gene tree not matching the unbalanced species tree. 6
S3 Scenarios for unrooted gene tree matching the balanced species tree. 13
S4 Scenarios for unrooted gene tree not matching the balanced species tree. 14
S5 Root branch calculations based on balanced or unbalanced quartets. 17
S6 Illustration of counters and their recursive formulas. 22
S7 Illustration of recursive formulas (continued). 23
S8 Mean absolute error on terminal and internal branches for quartet datasets. 28
S9 Mean absolute error on terminal and internal branches for S100 datasets. 28
S10 Mean absolute and log error on terminal and internal branches for MVRoot datasets. . . . 29
S11 Root mean squared error (RMSE) and log error on the quartet datasets. 30
S12 Correlations between true and estimated branch lengths on the quartet datasets. 31
S13 Correlations between true and estimated branch lengths on the S100 dataset. 31
S14 Root mean squared error (RMSE) and log error on the S100 datasets. 32
S15 Runtime and peak memory usage on the S100 datasets. 33
S16 Root mean squared error (RMSE) and bias on the MVRoot datasets. 34
S17 Correlation between ILS and log error on the MVRoot datasets. 35
S18 Mean absolute error and mean log error on the MVRoot datasets. 36
S19 Visualization of example simulated and biological trees 37
S20 Correlations between CASTLES and CA-ML branch lengths on the mammalian dataset. . 38

List of Tables

S1 Summary of exact formulas for expected branch lengths in gene trees. 17
S2 Summary of simplified formulas for expected branch lengths in gene trees. 18
S3 Summary of formulas for estimating species tree branch lengths in SU. 18
S4 Details of the dynamic programming algorithm for calculating quartet statistics. 20
S5 Parameters used in SimPhy quartet simulations for all model conditions. 24
S6 Characteristics of the quartet simulation model conditions. 25

S1 Proofs for Branch Length Equations

Preliminaries. Under the MSC, waiting times before coalescent events are exponential random variables with rate $\lambda = \binom{k}{2}$, where k is the number of lineages entering an interval (Kingman, 1982). Therefore, the probability density function for the coalescence event between k lineages in an interval with length x is $f_X(x) = \lambda e^{-\lambda x} = \binom{k}{2} e^{-\binom{k}{2}x}$; i.e., e^{-x} for two lineages, $3e^{-3x}$ for three lineages, and $6e^{-6x}$ for four lineages. The mean of this random variable is $\frac{1}{\lambda} = \frac{1}{\binom{k}{2}}$ which is 1 and $\frac{1}{3}$ for two and three lineages respectively. Tavaré (1984) derived an equation for the function $g_{ij}(T)$ as the probability that i lineages coalesce into j lineages in time T. Specific cases of this function (shown in Figures S1 to S4) are tabulated by Rosenberg (2002) and can be used to double-check our derivations.

Notations. As shown in Figures S1 and S2, we assume an unbalanced model species tree $(((A, B) : T_1, C) : T_2, D)$ and a gene tree on the same leafset with an unrooted topology that either matches or does not match the topology of the species tree. In Figures S3 and S4, we work with the balanced species tree $((A, B) : T_1, (C, D) : T_2)$. The parameters of the model species tree (i.e. μ_is and T_is) are defined according to Figure 1 of the main paper. We denote the expected length of the internal branch in a gene tree with unrooted topology ψ (where ψ is either $ab|cd, ac|bd$ or $ad|bc$) by $\mathbb{E}(l_I(\psi))$, and the expected length of a terminal branch leading to taxa X by $\mathbb{E}(l_X(\psi))$. Figures S2 and S4 only show scenarios for non-matching gene trees that have the topology $ad|bc$, as the derivations for the other non-matching topology ($ac|bd$) is similar, and in all cases except for cherry branches, the expected lengths of a branch in these two topologies are the same.

We first compute the average expected length of the internal and terminal branches in a quartet gene tree for gene trees matching the topology of the species tree (denoted by L_I for the internal branch, L_X for the terminal branch leading to taxa X) as well as gene trees not matching the species tree (denoted by L'_I for internal branch, L'_X for terminal branch leading to taxa X) for both unbalanced and balanced model species trees.

To calculate these expected lengths, we consider all scenarios that lead to different branch lengths in matching or non-matching gene trees (summarized in Figures S1 to S4 for the internal branch) and their corresponding probabilities, and compute the following conditional expectation for the internal branch (similar expectations can be written for terminal branches, modifying l_I to l_X)

$$\mathbb{E}(l_I(\psi)) = \mathbb{E}(l_I|\Psi = \psi) = \int x f_{l_I|\Psi}(x|\psi)dx = \frac{1}{\mathbb{P}(\psi)} \int x f_{l_I,\Psi}(x, \psi)dx$$

where Ψ is a random variable denoting the unrooted gene tree topology, and $\mathbb{P}(\psi)$ is the probability of the specific topology ψ under the MSC, which can be computed as follows (Allman et al., 2011) for the unbalanced and balanced model species trees of Figure 1 respectively.

$$\text{unbalanced:} \quad \mathbb{P}(ab|cd) = 1 - \frac{2}{3}e^{-T_1} \quad , \quad \mathbb{P}(ac|bd) = \mathbb{P}(ad|bc) = \frac{1}{3}e^{-T_1}$$
$$\text{balanced:} \quad \mathbb{P}(ab|cd) = 1 - \frac{2}{3}e^{-(T_1+T_2)} \quad , \quad \mathbb{P}(ac|bd) = \mathbb{P}(ad|bc) = \frac{1}{3}e^{-(T_1+T_2)}$$

In some figures, one shape corresponds to more than one scenario (specified in the caption); we use this only when both scenarios give the same expected internal branch length and are derived by swapping two lineages. All the calculations (in particular, integrals) in the proofs are verified using Mathematica, and the notebook is available at https://github.com/ytabatabaee/CASTLES/blob/main/su_branch_calcs.nb.

S1.1 Unbalanced species trees (Theorem 1 and related lemmas)

Before proving Theorem 1, we introduce and prove four lemmas: All of our results and their proofs are in reference to Figures S1 and S2.

Lemma S1 (Internal unbalanced)**.** *For the unbalanced model species tree of Figure 1, the expected length of the internal branch of a gene tree with an unrooted topology matching the species tree in substitution units is*

$$L_I = \mathbb{E}(l_I(ab|cd)) = \frac{(e^{-3T_2} + 3e^{-T_2} - 6e^{T_1-T_2})(\mu_2 - \mu_3) + 6(1 - e^{T_1} + T_1 e^{T_1})\mu_1}{2(3e^{T_1} - 2)} + \mu_2 \tag{S1}$$

and the expected length for gene trees not matching the species tree topology is

$$L'_I = \mathbb{E}(l_I(ac|bd)) = \mathbb{E}(l_I(ad|bc)) = \mu_2 + \frac{1}{2}(\mu_2 - \mu_3)\left(e^{-3T_2} - 3e^{-T_2}\right) . \tag{S2}$$

Lemma S2 (Terminal A or B (cherries), unbalanced)**.** *For the unbalanced model species tree of Figure 1, the expected length of the terminal edge A (equivalently, B) of a gene tree with an unrooted topology matching the species tree in substitution units is*

$$L_A = \mathbb{E}(l_A(ab|cd)) = \frac{6T_1\mu_1 + 3\mu_1 - \mu_2 + e^{-3T_2}(\mu_2 - 2\mu_3)}{6 - 9e^{T_1}} + \mu_1 + \mu_A T_A \tag{S3}$$

and the expected lengths for gene trees not matching the species tree topology are

$$\mathbb{E}(l_A(ad|bc)) = \left(\frac{1}{6}e^{-3T_2} - \frac{3}{2}e^{-T_2}\right)(\mu_2 - \mu_3) - \frac{2}{3}e^{-3T_2}\mu_3 + \frac{4}{3}\mu_2 + T_1\mu_1 + T_A\mu_A$$
$$\mathbb{E}(l_A(ac|bd)) = -\frac{1}{3}e^{-3T_2}(\mu_2 - 2\mu_3) + \frac{1}{3}\mu_2 + T_1\mu_1 + T_A\mu_A$$

and therefore

$$L'_A = \frac{1}{2}(\mathbb{E}(l_A(ad|bc)) + \mathbb{E}(l_A(ac|bd))) = \frac{1}{12}(10\mu_2 - 9e^{-T_2}(\mu_2 - \mu_3) - 3^{-3T_2}(\mu_2 + \mu_3)) + T_1\mu_1 + T_A\mu_A \tag{S4}$$

Lemma S3 (Terminal unbalanced C)**.** *For the unbalanced species tree, the expected length of the terminal edge C of a gene tree with an unrooted topology matching the species tree in substitution units is*

$$L_C = \mathbb{E}(l_C(ab|cd)) = -\,e^{-T_2}(\mu_2 - \mu_3) + \mu_2 + \mu_C T_C + \frac{2\mu_2 - \left(3e^{-T_2} - e^{-3T_2}\right)(\mu_2 - \mu_3) - 4\mu_3 e^{-3T_2}}{6(3e^{T_1} - 2)} \tag{S5}$$

and the expected length for gene trees not matching the species tree topology is

$$L'_C = \mathbb{E}(l_C(ac|bd)) = \frac{1}{3}\mu_2(1 + e^{-3T_2}) + \mu_C T_C \tag{S6}$$

Lemma S4 (Terminal unbalanced D)**.** *For the unbalanced species tree, the expected length of the terminal edge D of a gene tree with an unrooted topology matching the species tree in substitution units is*

$$L_D = \mathbb{E}(l_D(ab|cd)) = e^{-T_2}(\mu_2 - \mu_3) - \mu_2 + 2\mu_3 + T_2\mu_2 + \mu_D T_D + \frac{-2\mu_2 + \left(3e^{-T_2} - e^{-3T_2}\right)(\mu_2 - \mu_3)}{6(3e^{T_1} - 2)} \tag{S7}$$

and the expected length for gene trees not matching the species tree topology is

$$L'_D = \mathbb{E}(l_D(ad|cb)) = \left(\frac{3}{2}e^{-T_2} - \frac{1}{6}e^{-3T_2}\right)(\mu_2 - \mu_3) - \frac{4}{3}\mu_2 + 2\mu_3 + T_2\mu_2 + \mu_D T_D \tag{S8}$$

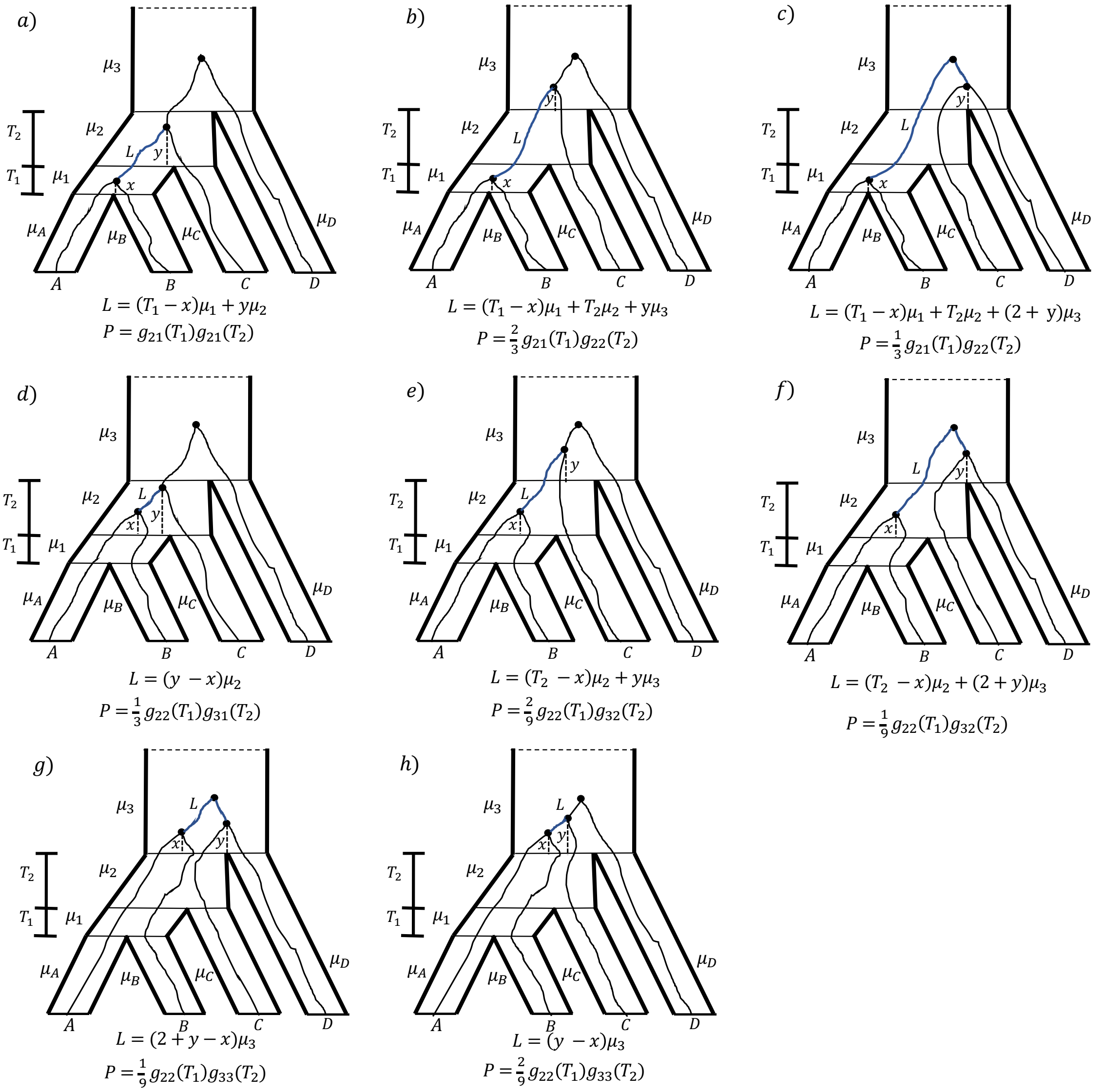

Figure S1: **Scenarios for gene tree matching the unbalanced species tree.** Internal branch lengths for unrooted quartet gene tree matching the unbalanced model species tree $(((A, B) : T_1, C) : T_2, D)$. L denotes the internal branch length in the gene tree and P denotes the probability of each case. Case (b) and (e) correspond to two scenarios and case (h) corresponds to four different scenarios, and the P values reported in these cases show the overall probability of all possible scenarios for that case.

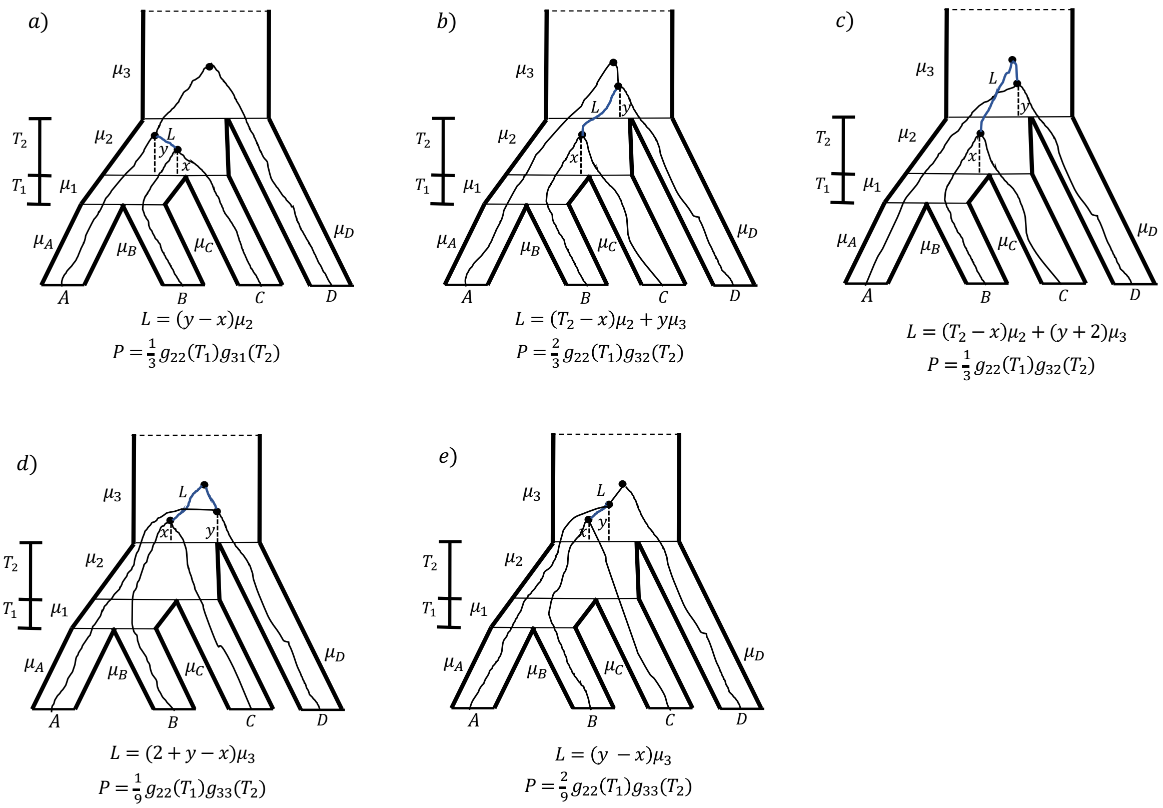

Figure S2: **Scenarios for gene tree not matching the unbalanced species tree.** Internal branch lengths for unrooted quartet gene tree not matching the unbalanced model species tree $(((A, B) : T_1, C) : T_2, D)$. L denotes the internal branch length in the gene tree and P denotes the probability of each case. Case (b) and (d) correspond to two scenarios and case (e) corresponds to four different scenarios, and the P values reported in these cases show the overall probability of all possible scenarios for that case.

Proof of Lemma S1. Referring to Figure S1, we can compute

$$
\begin{aligned}
\mathbb{E}(L_I(ab|cd)) = \Bigg(& \int_0^{T_1}\int_0^{T_2} e^{-x}e^{-y}((T_1-x)\mu_1+y\mu_2)\,dy\,dx && \text{(scenario (a))}\\
& + 2e^{-T_2}\int_0^{T_1}\int_0^{\infty} e^{-x}3e^{-3y}\frac{}{3}((T_1-x)\mu_1+T_2\mu_2+y\mu_3)\,dy\,dx && \text{(scenario (b))}\\
& + e^{-T_2}\int_0^{T_1}\int_0^{\infty} e^{-x}3e^{-3y}\frac{1}{3}((T_1-x)\mu_1+T_2\mu_2+(2+y)\mu_3)\,dy\,dx && \text{(scenario (c))}\\
& + e^{-T_1}\int_0^{T_2}\int_x^{T_2} 3e^{-3x}e^{-(y-x)}\frac{1}{3}(y-x)\mu_2\,dy\,dx && \text{(scenario (d))}\\
& + 2e^{-T_1}\int_0^{T_2}\int_0^{\infty} 3e^{-3x}e^{-(T_2-x)}3e^{-3y}\frac{1}{3\times 3}((T_2-x)\mu_2+y\mu_3)\,dy\,dx && \text{(scenario (e))}\\
& + e^{-T_1}\int_0^{T_2}\int_0^{\infty} 3e^{-3x}e^{-(T_2-x)}3e^{-3y}\frac{1}{3\times 3}((T_2-x)\mu_2+(2+y)\mu_3)\,dy\,dx && \text{(scenario (f))}\\
& + 2e^{-T_1}e^{-3T_2}\int_0^{\infty}\int_x^{\infty} 6e^{-6x}3e^{-3(y-x)}\frac{1}{6\times 3}(2+y-x)\mu_3\,dy\,dx && \text{(scenario (g))}\\
& +4e^{-T_1}e^{-3T_2}\int_0^{\infty}\int_x^{\infty} 6e^{-6x}3e^{-3(y-x)}\frac{1}{6\times 3}(y-x)\mu_3\,dy\,dx\Bigg) \Big/ \left(1-\frac{2}{3}e^{-T_1}\right) && \text{(scenario (h))}\\
& = \frac{(e^{-3T_2}+3e^{-T_2}-6e^{T_1-T_2})(\mu_2-\mu_3)+6(1-e^{T_1}+T_1e^{T_1})\mu_1}{2(3e^{T_1}-2)}+\mu_2 && \text{(S9)}
\end{aligned}
$$

Similarly, referring to Figure S2, we can compute

$$
\begin{aligned}
\mathbb{E}(L_I(ac|bd)) = \Bigg(& e^{-T_1}\int_0^{T_2}\int_x^{T_2} 3e^{-3x}e^{-(y-x)}\frac{1}{3}(y-x)\mu_2\,dy\,dx && \text{(scenario (a))}\\
& + 2e^{-T_1}\int_0^{T_2}\int_0^{\infty} 3e^{-3x}e^{-(T_2-x)}3e^{-3y}\frac{1}{3\times 3}((T_2-x)\mu_2+y\mu_3)\,dy\,dx && \text{(scenario (b))}\\
& + e^{-T_1}\int_0^{T_2}\int_0^{\infty} 3e^{-3x}e^{-(T_2-x)}3e^{-3y}\frac{1}{3\times 3}((T_2-x)\mu_2+(2+y)\mu_3)\,dy\,dx && \text{(scenario (c))}\\
& + 2e^{-T_1}e^{-3T_2}\int_0^{\infty}\int_x^{\infty} 6e^{-6x}3e^{-3(y-x)}\frac{1}{6\times 3}(2+y-x)\mu_3\,dy\,dx && \text{(scenario (d))}\\
& +4e^{-T_1}e^{-3T_2}\int_0^{\infty}\int_x^{\infty} 6e^{-6x}3e^{-3(y-x)}\frac{1}{6\times 3}(y-x)\mu_3\,dy\,dx\Bigg) \Big/ \left(\frac{1}{3}e^{-T_1}\right) && \text{(scenario (e))}\\
& = \mu_2+\frac{1}{2}(\mu_2-\mu_3)\left(e^{-3T_2}-3e^{-T_2}\right) && \text{(S10)}
\end{aligned}
$$

□

Proof of Lemma S2. Referring to Figure S1, we can compute

$$\mathbb{E}(L_A(ab|cd)) = \Bigg(\int_0^{T_1} e^{-x}(x\mu_1 + T_A\mu_A)\,dx \qquad \text{(scenario (a) to (c))}$$

$$+ e^{-T_1}\int_0^{T_2}\int_x^{T_2} 3e^{-3x}e^{-(y-x)}\frac{1}{3}(x\mu_2 + T_1\mu_1 + T_A\mu_A)\,dy\,dx \qquad \text{(scenario (d))}$$

$$+ 3e^{-T_1}\int_0^{T_2}\int_0^{\infty} 3e^{-3x}e^{-(T_2-x)}3e^{-3y}\frac{1}{3\times 3}(x\mu_2 + T_1\mu_1 + T_A\mu_A)\,dy\,dx \qquad \text{(scenario (e), (f))}$$

$$+ 2e^{-T_1}e^{-3T_2}\int_0^{\infty}\int_x^{\infty} 6e^{-6x}3e^{-3(y-x)}\frac{1}{6\times 3}(y\mu_3 + T_2\mu_2 + T_1\mu_1 + T_A\mu_A)\,dy\,dx \qquad \text{(scenario (g), (h))}$$

$$+ e^{-T_1}e^{-3T_2}\int_0^{\infty}\int_x^{\infty} 6e^{-6x}3e^{-3(y-x)}\frac{1}{6\times 3}((y+2)\mu_3 + T_2\mu_2 + T_1\mu_1 + T_A\mu_A)\,dy\,dx \qquad \text{(scenario (h))}$$

$$+3e^{-T_1}e^{-3T_2}\int_0^{\infty}\int_x^{\infty} 6e^{-6x}3e^{-3(y-x)}\frac{1}{6\times 3}(x\mu_3 + T_2\mu_2 + T_1\mu_1 + T_A\mu_A)\,dy\,dx\Bigg) \Big/ \left(1 - \frac{2}{3}e^{-T_1}\right) \qquad \text{(scenario (g), (h))}$$

$$= \frac{6T_1\mu_1 + 3\mu_1 - \mu_2 + e^{-3T_2}(\mu_2 - 2\mu_3)}{6 - 9e^{T_1}} + \mu_1 + \mu_A T_A \qquad \text{(S11)}$$

Referring to Figure S2, we can compute

$$\mathbb{E}(L_A(ad|bc)) = \Bigg(e^{-T_1}\int_0^{T_2}\int_x^{T_2} 3e^{-3x}e^{-(y-x)}\frac{1}{3}(y\mu_2 + T_1\mu_1 + T_A\mu_A)\,dy\,dx \qquad \text{(scenario (a))}$$

$$+ e^{-T_1}\int_0^{T_2}\int_0^{\infty} 3e^{-3x}e^{-(T_2-x)}3e^{-3y}\frac{1}{3\times 3}((y+2)\mu_3 + T_2\mu_2 + T_1\mu_1 + T_A\mu_A)\,dy\,dx \qquad \text{(scenario (b))}$$

$$+ 2e^{-T_1}\int_0^{T_2}\int_0^{\infty} 3e^{-3x}e^{-(T_2-x)}3e^{-3y}\frac{1}{3\times 3}(y\mu_3 + T_2\mu_2 + T_1\mu_1 + T_A\mu_A)\,dy\,dx \qquad \text{(scenario (b), (c))}$$

$$+ 2e^{-T_1}e^{-3T_2}\int_0^{\infty}\int_x^{\infty} 6e^{-6x}3e^{-3(y-x)}\frac{1}{6\times 3}(y\mu_3 + T_2\mu_2 + T_1\mu_1 + T_A\mu_A)\,dy\,dx \qquad \text{(scenario (d), (e))}$$

$$+ e^{-T_1}e^{-3T_2}\int_0^{\infty}\int_x^{\infty} 6e^{-6x}3e^{-3(y-x)}\frac{1}{6\times 3}((y+2)\mu_3 + T_2\mu_2 + T_1\mu_1 + T_A\mu_A)\,dy\,dx \qquad \text{(scenario (e))}$$

$$+3e^{-T_1}e^{-3T_2}\int_0^{\infty}\int_x^{\infty} 6e^{-6x}3e^{-3(y-x)}\frac{1}{6\times 3}(x\mu_3 + T_2\mu_2 + T_1\mu_1 + T_A\mu_A)\,dy\,dx\Bigg) \Big/ \left(\frac{1}{3}e^{-T_1}\right) \qquad \text{(scenario (d), (e))}$$

$$= \left(\frac{1}{6}e^{-3T_2} - \frac{3}{2}e^{-T_2}\right)(\mu_2 - \mu_3) - \frac{2}{3}e^{-3T_2}\mu_3 + \frac{4}{3}\mu_2 + T_1\mu_1 + T_A\mu_A \qquad \text{(S12)}$$

and

$$\mathbb{E}(L_A(ac|bd)) = \Bigg(e^{-T_1}\int_0^{T_2}\int_x^{T_2} 3e^{-3x}e^{-(y-x)}\frac{1}{3}(x\mu_2+T_1\mu_1+T_A\mu_A)\,dy\,dx \qquad \text{(scenario (a))}$$

$$+\,3e^{-T_1}\int_0^{T_2}\int_0^{\infty} 3e^{-3x}e^{-(T_2-x)}3e^{-3y}\frac{1}{3\times 3}(x\mu_2+T_1\mu_1+T_A\mu_A)\,dy\,dx \qquad \text{(scenario (b), (c))}$$

$$+\,2e^{-T_1}e^{-3T_2}\int_0^{\infty}\int_x^{\infty} 6e^{-6x}3e^{-3(y-x)}\frac{1}{6\times 3}(y\mu_3+T_2\mu_2+T_1\mu_1+T_A\mu_A)\,dy\,dx \qquad \text{(scenario (d), (e))}$$

$$+\,e^{-T_1}e^{-3T_2}\int_0^{\infty}\int_x^{\infty} 6e^{-6x}3e^{-3(y-x)}\frac{1}{6\times 3}((y+2)\mu_3+T_2\mu_2+T_1\mu_1+T_A\mu_A)\,dy\,dx \qquad \text{(scenario (e))}$$

$$+3e^{-T_1}e^{-3T_2}\int_0^{\infty}\int_x^{\infty} 6e^{-6x}3e^{-3(y-x)}\frac{1}{6\times 3}(x\mu_3+T_2\mu_2+T_1\mu_1+T_A\mu_A)\,dy\,dx\Bigg)\Big/\left(\frac{1}{3}e^{-T_1}\right) \qquad \text{(scenario (d), (e))}$$

$$= -\frac{1}{3}e^{-3T_2}(\mu_2-2\mu_3)+\frac{1}{3}\mu_2+T_1\mu_1+T_A\mu_A \tag{S13}$$

□

Proof of Lemma S3. Referring to Figure S1, we can compute

$$\mathbb{E}(L_C(ab|cd)) = \Bigg(\int_0^{T_1}\int_0^{T_2} e^{-x}e^{-y}(y\mu_2+T_C\mu_C)\,dy\,dx \qquad \text{(scenario (a))}$$

$$+\,e^{-T_2}\int_0^{T_1}\int_0^{\infty} e^{-x}3e^{-3y}\frac{1}{3}((y+2)\mu_3+T_2\mu_2+T_C\mu_C)\,dy\,dx \qquad \text{(scenario (b))}$$

$$+\,2e^{-T_2}\int_0^{T_1}\int_0^{\infty} e^{-x}3e^{-3y}\frac{1}{3}(y\mu_3+T_2\mu_2+T_C\mu_C)\,dy\,dx \qquad \text{(scenario (b), (c))}$$

$$+\,e^{-T_1}\int_0^{T_2}\int_x^{T_2} 3e^{-3x}e^{-(y-x)}\frac{1}{3}(y\mu_2+T_C\mu_C)\,dy\,dx \qquad \text{(scenario (d))}$$

$$+\,e^{-T_1}\int_0^{T_2}\int_0^{\infty} 3e^{-3x}e^{-(T_2-x)}3e^{-3y}\frac{1}{3\times 3}((y+2)\mu_3+T_2\mu_2+T_C\mu_C)\,dy\,dx \qquad \text{(scenario (e))}$$

$$+\,2e^{-T_1}\int_0^{T_2}\int_0^{\infty} 3e^{-3x}e^{-(T_2-x)}3e^{-3y}\frac{1}{3\times 3}(y\mu_3+T_2\mu_2+T_C\mu_C)\,dy\,dx \qquad \text{(scenario (e), (f))}$$

$$+\,2e^{-T_1}e^{-3T_2}\int_0^{\infty}\int_x^{\infty} 6e^{-6x}3e^{-3(y-x)}\frac{1}{6\times 3}(y\mu_3+T_2\mu_2+T_C\mu_C)\,dy\,dx \qquad \text{(scenario (g), (h))}$$

$$+\,e^{-T_1}e^{-3T_2}\int_0^{\infty}\int_x^{\infty} 6e^{-6x}3e^{-3(y-x)}\frac{1}{6\times 3}((y+2)\mu_3+T_2\mu_2+T_C\mu_C)\,dy\,dx \qquad \text{(scenario (h))}$$

$$+3e^{-T_1}e^{-3T_2}\int_0^{\infty}\int_x^{\infty} 6e^{-6x}3e^{-3(y-x)}\frac{1}{6\times 3}(x\mu_3+T_2\mu_2+T_C\mu_C)\,dy\,dx\Bigg)\Big/\left(1-\frac{2}{3}e^{-T_1}\right) \qquad \text{(scenario (g), (h))}$$

$$= -e^{-T_2}(\mu_2-\mu_3)+\frac{2\mu_2-\left(3e^{-T_2}-e^{-3T_2}\right)(\mu_2-\mu_3)-4\mu_3e^{-3T_2}}{6(3e^{T_1}-2)}+\mu_2+\mu_C T_C \tag{S14}$$

And referring to Figure S2, we can compute

$$
\begin{aligned}
\mathbb{E}(L_C(ac|bd)) = \mathbb{E}(L_C(ad|bc)) = \\
&= \Bigg(e^{-T_1} \int_0^{T_2} \int_x^{T_2} 3e^{-3x} e^{-(y-x)} \frac{1}{3} (x\mu_2 + T_C\mu_C) \, dy \, dx && \text{(scenario (a))} \\
&+ 3e^{-T_1} \int_0^{T_2} \int_0^{\infty} 3e^{-3x} e^{-(T_2-x)} 3e^{-3y} \frac{1}{3} \times \frac{1}{3} (x\mu_2 + T_C\mu_C) \, dy \, dx && \text{(scenario (b), (c))} \\
&+ 2e^{-T_1} e^{-3T_2} \int_0^{\infty} \int_x^{\infty} 6e^{-6x} 3e^{-3(y-x)} \frac{1}{6 \times 3} (y\mu_3 + T_2\mu_2 + T_C\mu_C) \, dy \, dx \\
& && \text{(scenario (d), (e))} \\
&+ e^{-T_1} e^{-3T_2} \int_0^{\infty} \int_x^{\infty} 6e^{-6x} 3e^{-3(y-x)} \frac{1}{6 \times 3} ((y+2)\mu_3 + T_2\mu_2 + T_C\mu_C) \, dy \, dx \\
& && \text{(scenario (e))} \\
&+ 3e^{-T_1} e^{-3T_2} \int_0^{\infty} \int_x^{\infty} 6e^{-6x} 3e^{-3(y-x)} \frac{1}{6 \times 3} (x\mu_3 + T_2\mu_2 + T_C\mu_C) \, dy \, dx \Bigg) \Big/ \left(\frac{1}{3} e^{-T_1} \right) \\
& && \text{(scenario (d), (e))} \\
&= \frac{1}{3} \mu_2 (1 + e^{-3T_2}) + \mu_C T_C && \text{(S15)}
\end{aligned}
$$

□

Proof of Lemma S4. Referring to Figure S1, we can compute

$$
\begin{aligned}
\mathbb{E}(L_D(ab|cd)) &= \Bigg(\int_0^{T_1} \int_0^{T_2} e^{-x} e^{-y} ((T_2 - y)\mu_2 + 2\mu_3 + T_D\mu_D) \, dy \, dx && \text{(scenario (a))} \\
&+ e^{-T_2} \int_0^{T_1} \int_0^{\infty} e^{-x} 3e^{-3y} \frac{1}{3} ((y+2)\mu_3 + T_D\mu_D) \, dy \, dx && \text{(scenario (b))} \\
&+ 2e^{-T_2} \int_0^{T_1} \int_0^{\infty} e^{-x} 3e^{-3y} \frac{1}{3} (y\mu_3 + T_D\mu_D) \, dy \, dx && \text{(scenario (b), (c))} \\
&+ e^{-T_1} \int_0^{T_2} \int_x^{T_2} 3e^{-3x} e^{-(y-x)} \frac{1}{3} ((T_2 - y)\mu_2 + 2\mu_3 + T_D\mu_D) \, dy \, dx && \text{(scenario (d))} \\
&+ e^{-T_1} \int_0^{T_2} \int_0^{\infty} 3e^{-3x} e^{-(T_2-x)} 3e^{-3y} \frac{1}{3 \times 3} ((y+2)\mu_3 + T_D\mu_D) \, dy \, dx && \text{(scenario (e))} \\
&+ 2e^{-T_1} \int_0^{T_2} \int_0^{\infty} 3e^{-3x} e^{-(T_2-x)} 3e^{-3y} \frac{1}{3 \times 3} (y\mu_3 + T_D\mu_D) \, dy \, dx && \text{(scenario (e), (f))} \\
&+ 2e^{-T_1} e^{-3T_2} \int_0^{\infty} \int_x^{\infty} 6e^{-6x} 3e^{-3(y-x)} \frac{1}{6 \times 3} (y\mu_3 + T_D\mu_D) \, dy \, dx && \text{(scenario (g), (h))} \\
&+ e^{-T_1} e^{-3T_2} \int_0^{\infty} \int_x^{\infty} 6e^{-6x} 3e^{-3(y-x)} \frac{1}{6 \times 3} ((y+2)\mu_3 + T_D\mu_D) \, dy \, dx && \text{(scenario (h))} \\
&+ 3e^{-T_1} e^{-3T_2} \int_0^{\infty} \int_x^{\infty} 6e^{-6x} 3e^{-3(y-x)} \frac{1}{6 \times 3} (x\mu_3 + T_D\mu_D) \, dy \, dx \Bigg) \Big/ \left(1 - \frac{2}{3} e^{-T_1} \right) \\
& && \text{(scenario (g), (h))} \\
&= e^{-T_2}(\mu_2 - \mu_3) + \frac{-2\mu_2 + \left(3e^{-T_2} - e^{-3T_2}\right)(\mu_2 - \mu_3)}{6(3e^{T_1} - 2)} - \mu_2 + 2\mu_3 + T_2\mu_2 + \mu_D T_D && \text{(S16)}
\end{aligned}
$$

Referring to Figure S2, we can compute

$$
\begin{aligned}
&\mathbb{E}(L_D(ac|bd)) = \mathbb{E}(L_D(ad|bc)) = \\
&= \Bigg(e^{-T_1} \int_0^{T_2} \int_x^{T_2} 3e^{-3x} e^{-(y-x)} \frac{1}{3}((T_2 - y)\mu_2 + 2\mu_3 + T_D\mu_D)\, dy\, dx && \text{(scenario (a))} \\
&+ e^{-T_1} \int_0^{T_2} \int_0^{\infty} 3e^{-3x} e^{-(T_2-x)} 3e^{-3y} \frac{1}{3\times 3}((y+2)\mu_3 + T_D\mu_D)\, dy\, dx && \text{(scenario (b))} \\
&+ 2e^{-T_1} \int_0^{T_2} \int_0^{\infty} 3e^{-3x} e^{-(T_2-x)} 3e^{-3y} \frac{1}{3\times 3}(y\mu_3 + T_D\mu_D)\, dy\, dx && \text{(scenario (b), (c))} \\
&+ 2e^{-T_1} e^{-3T_2} \int_0^{\infty} \int_x^{\infty} 6e^{-6x} 3e^{-3(y-x)} \frac{1}{6\times 3}(y\mu_3 + T_D\mu_D)\, dy\, dx && \text{(scenario (d), (e))} \\
&+ e^{-T_1} e^{-3T_2} \int_0^{\infty} \int_x^{\infty} 6e^{-6x} 3e^{-3(y-x)} \frac{1}{6\times 3}((y+2)\mu_3 + T_D\mu_D)\, dy\, dx && \text{(scenario (e))} \\
&+3e^{-T_1} e^{-3T_2} \int_0^{\infty} \int_x^{\infty} 6e^{-6x} 3e^{-3(y-x)} \frac{1}{6\times 3}(x\mu_3 + T_D\mu_D)\, dy\, dx \Bigg) \Big/ \left(\frac{1}{3}e^{-T_1}\right) \\
& && \text{(scenario (d), (e))} \\
&= \left(\frac{3}{2}e^{-T_2} - \frac{1}{6}e^{-3T_2}\right)(\mu_2 - \mu_3) - \frac{4}{3}\mu_2 + 2\mu_3 + T_2\mu_2 + \mu_D T_D && \text{(S17)}
\end{aligned}
$$

□

With these lemmas, we can now prove the main results.

Proof of Theorem 1. Subtracting Eq. S10 from Eq. S9, we get

$$\Delta_I = \mathbb{E}(L_I(ab|cd)) - \mathbb{E}(L_I(ac|bd)) = \frac{3(e^{-T_2} - e^{-3T_2})(1 - e^{-T_1})(\mu_2 - \mu_3) + 6\mu_1(e^{-T_1} - 1 + T_1)}{2(3 - 2e^{-T1})} \tag{S18}$$

The average length of the terminal branch of A in a gene tree not matching the topology of the species tree is therefore the average of Eq. S12 and Eq. S13

$$\frac{1}{2}(\mathbb{E}(L_A(ad|bc)) + \mathbb{E}(L_A(ac|bd))) = \frac{1}{12}(10\mu_2 - 9e^{-T_2}(\mu_2 - \mu_3) - 3^{-3T_2}(\mu_2 + \mu_3)) + T_1\mu_1 + T_A\mu_A \tag{S19}$$

Subtracting Eq. S19 from Eq. S11, we get

$$\Delta_A = \mathbb{E}(L_A(ab|cd)) - \frac{1}{2}(\mathbb{E}(L_A(ad|bc)) + \mathbb{E}(L_A(ac|bd))) \tag{S20}$$

$$= \frac{\left(4\mu_2 - 6\mu_1 - (3e^{-T_2} + e^{-3T_2})(\mu_2 - \mu_3) + e^{T_1}\left(\frac{1}{2}(\mu_2 + \mu_3)e^{-3T_2} + \frac{9}{2}e^{-T_2}(\mu_2 - \mu_3) - 6T_1\mu_1 + 6\mu_1 - 5\mu_2\right)\right)}{2(-2 + 3e^{T_1})} \tag{S21}$$

Subtracting Eq. S15 from Eq. S14, we get

$$\Delta_C = \mathbb{E}(L_C(ab|cd)) - \mathbb{E}(L_C(ac|bd)) = \frac{(2 - e^{-T_1})\left(\left(e^{-3T_2} + 2\right)\mu_2 - 3e^{-T_2}(\mu_2 - \mu_3)\right) + \mu_3 e^{-3T_2}(e^{-T_1} - 4)}{2(3 - 2e^{-T_1})} \tag{S22}$$

Subtracting Eq. S17 from Eq. S16, we get

$$\Delta_D = \mathbb{E}(L_D(ab|cd)) - \mathbb{E}(L_D(ac|bd)) = \frac{(1 - e^{-T_1})(2\mu_2 - (3e^{-T_2} - e^{-3T_2})(\mu_2 - \mu_3))}{2(3 - 2e^{-T_1})} \tag{S23}$$

□

S1.2 Balanced Species Tree

Similar to Theorem 1 for unbalanced trees, we now provide Theorem S1 for balanced trees.

Theorem S1 (Balanced)**.** *For the balanced model species tree of Figure 1, let Δ_I be the difference in the expected internal branch length in substitution units of gene trees with an unrooted topology matching the species tree and those not matching the species tree. Then,*

$$\Delta_I = \frac{3(e^{-T_1}(\mu_1 - \mu_3) + \mu_3 e^{-(T_1+T_2)} + e^{-T_2}(\mu_2 - \mu_3) + (T_1\mu_1 + T_2\mu_2 - \mu_1 - \mu_2 + \mu_3))}{3 - 2e^{-(T_1+T_2)}} \tag{S24}$$

Similarly, let Δ_A be the difference in the expected length of matching and non-matching gene trees for the terminal branch leading to a cherry A.

$$\Delta_A = \frac{-\mu_3 e^{-(T_1+T_2)} + 3\mu_1(1 - e^{-T_1} - T_1) + \mu_3(-2 + 3e^{-T_1})}{3 - 2e^{-(T_1+T_2)}} \tag{S25}$$

Note that since all taxa in a balanced quartet tree are part of a cherry, B, C and D would follow similar equations as (S25) by substituting the appropriate μ and T values, following the symmetry of the tree. We first introduce and prove two lemmas, and then follow up with the proof of Theorem S1. All of our results and their proofs are in reference to Figures S3 and S4.

Lemma S5 (Internal balanced)**.** *For the unbalanced model species tree of Figure 1, the expected length of the internal branch of a gene tree with an unrooted topology matching the species tree in substitution units is*

$$L_I = \mathbb{E}(l_I(ab|cd)) = \frac{3e^{-T_1}(\mu_1 - \mu_3) + \mu_3 e^{-(T_1+T_2)} + 3e^{-T_2}(\mu_2 - \mu_3) + 3(T_1\mu_1 + T_2\mu_2 - \mu_1 - \mu_2 + 2\mu_3)}{3 - 2e^{-(T_1+T_2)}} \tag{S26}$$

and the expected length for gene trees not matching the species tree topology is

$$L'_I = \mathbb{E}(l_I(ac|bd)) = \mathbb{E}(l_I(ad|bc)) = \mu_3 \tag{S27}$$

Lemma S6 (Terminal (cherries), balanced)**.** *For the unbalanced model species tree of Figure 1, the expected length of the terminal edge A (equivalently, B) of a gene tree with an unrooted topology matching the species tree in substitution units is*

$$L_A = \mathbb{E}(l_A(ab|cd)) = \frac{e^{-(T_1+T_2)}(-6T_1\mu_1 - 7\mu_3) + 9((1 - e^{-T_1})\mu_1 + \mu_3 e^{-T_1})}{9 - 6e^{-(T_1+T_2)}} + \mu_A T_A \tag{S28}$$

and the expected lengths for gene trees not matching the species tree topology are

$$L'_A = \mathbb{E}(l_A(ad|bc)) = \mathbb{E}(l_A(ac|bd)) = T_1\mu_1 + \frac{2}{3}\mu_3 + \mu_A T_A \tag{S29}$$

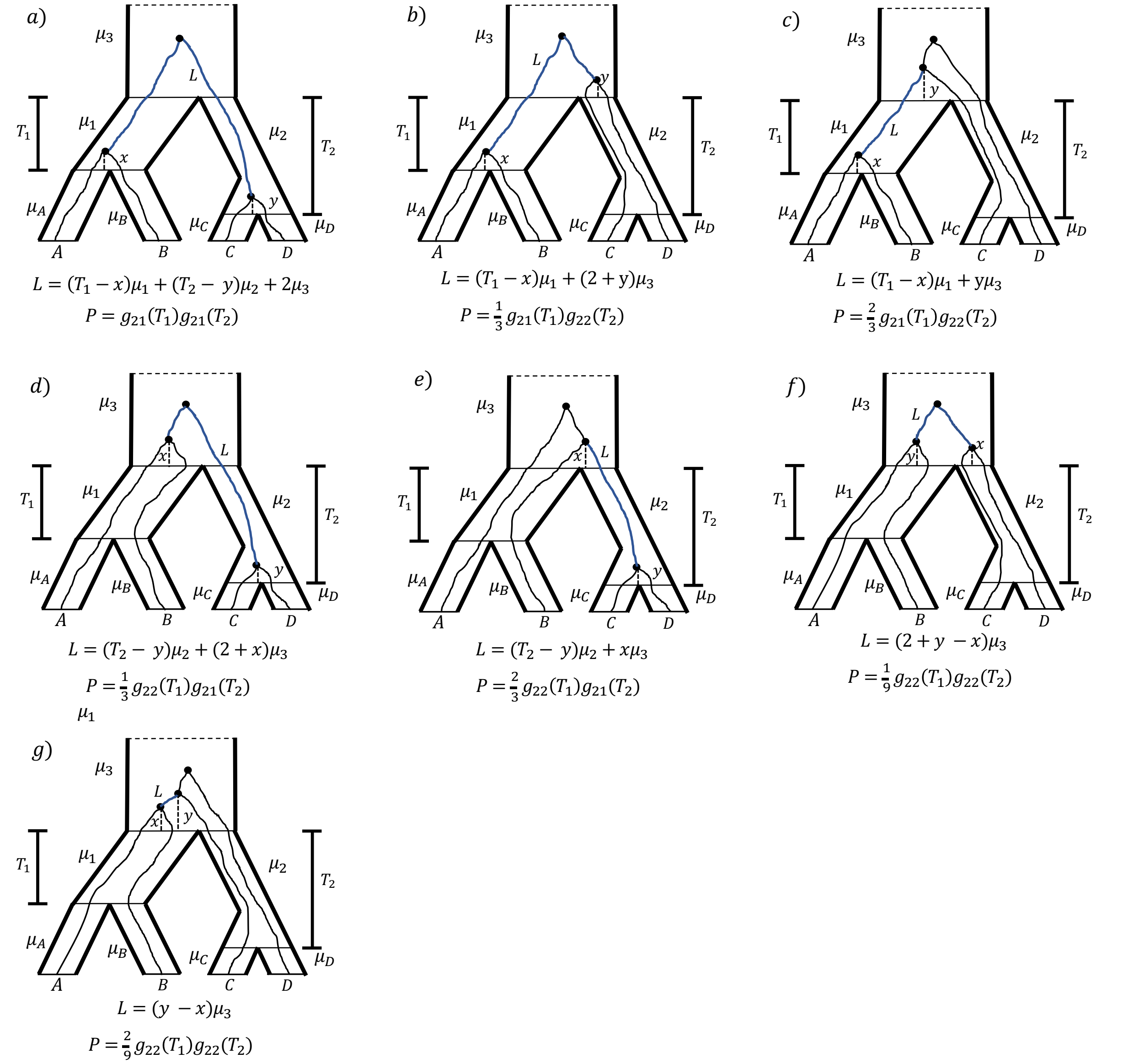

Figure S3: **Scenarios for gene tree matching the balanced species tree.** Internal branch lengths for unrooted quartet gene tree matching the balanced model species tree $((A, B) : T_1, (C, D) : T_2)$. Here, L denotes the internal branch length in the gene tree and P denotes the probability of each case. Case (c) and (e) correspond to two scenarios and case (g) corresponds to four different scenarios, and the P values reported in these cases show the overall probability of all possible scenarios for that case.

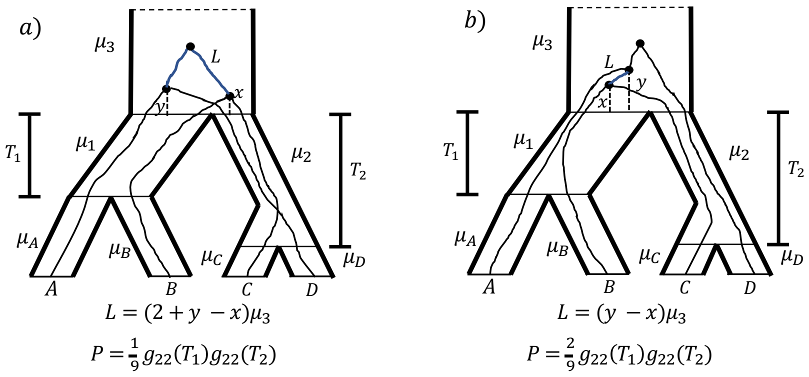

Figure S4: **Scenarios for gene tree not matching the balanced species tree.** Internal branch lengths for unrooted quartet gene tree not matching the balanced model species tree $((A,B):T_1,(C,D):T_2)$. L denotes the internal branch length in the gene tree and P denotes the probability of each case. Case (b) corresponds to four different scenarios.

Proof of Lemma S5. Referring to Figure S3, we can compute

$$\begin{aligned}
\mathbb{E}(L_I(ab|cd)) = &\Bigg(\int_0^{T_1}\int_0^{T_2} e^{-x}e^{-y}((T_1-x)\mu_1+(T_2-y)\mu_2+2\mu_3)\,dy\,dx && \text{(scenario (a))}\\
&+e^{-T_2}\int_0^{T_1}\int_0^{\infty} e^{-x}3e^{-3y}\frac{1}{3}((T_1-x)\mu_1+(2+y)\mu_3)\,dy\,dx && \text{(scenario (b))}\\
&+e^{-T_2}\int_0^{T_1}\int_0^{\infty} e^{-x}3e^{-3y}\frac{2}{3}((T_1-x)\mu_1+y\mu_3)\,dy\,dx && \text{(scenario (c))}\\
&+e^{-T_1}\int_0^{\infty}\int_0^{T_2} 3e^{-3x}e^{-y}\frac{1}{3}((T_2-y)\mu_2+(2+x)\mu_3)\,dy\,dx && \text{(scenario (d))}\\
&+e^{-T_1}\int_0^{\infty}\int_0^{T_2} 3e^{-3x}e^{-y}\frac{2}{3}((T_2-y)\mu_2+x\mu_3)\,dy\,dx && \text{(scenario (e))}\\
&+2e^{-T_1}e^{-T_2}\int_0^{\infty}\int_x^{\infty} 6e^{-6x}3e^{-3(y-x)}\frac{1}{6\times 3}(2+y-x)\mu_3\,dy\,dx && \text{(scenario (f))}\\
&+4e^{-T_1}e^{-T_2}\int_0^{\infty}\int_x^{\infty} 6e^{-6x}3e^{-3(y-x)}\frac{1}{6\times 3}(y-x)\mu_3\,dy\,dx\Bigg)\Big/\left(1-\frac{2}{3}e^{-(T_1+T_2)}\right)\\
& && \text{(scenario (g))}\\
&=\frac{3e^{-T_1}(\mu_1-\mu_3)+\mu_3e^{-(T_1+T_2)}+3e^{-T_2}(\mu_2-\mu_3)+3(T_1\mu_1+T_2\mu_2-\mu_1-\mu_2+2\mu_3)}{3-2e^{-(T_1+T_2)}}
\end{aligned} \tag{S30}$$

For a balanced model tree, there are only two scenarios for a gene tree not matching the species tree, shown in Figure S4. The expected length of the internal branch in this case is

$$\begin{aligned}
&\mathbb{E}(L_I(ad|bc)) = \mathbb{E}(L_I(ac|bd)) =\\
&\quad\Bigg(2e^{-T_1}e^{-T_2}\int_0^{\infty}\int_x^{\infty} 6e^{-6x}3e^{-3(y-x)}\frac{1}{6\times 3}(2+y-x)\mu_3\,dy\,dx && \text{(scenario (a))}\\
&\quad+4e^{-T_1}e^{-T_2}\int_0^{\infty}\int_x^{\infty} 6e^{-6x}3e^{-3(y-x)}\frac{1}{6\times 3}(y-x)\mu_3\,dy\,dx\Bigg)\Big/\left(\frac{1}{3}e^{-(T_1+T_2)}\right)\\
& && \text{(scenario (b))}\\
&\quad=\mu_3
\end{aligned} \tag{S31}$$

□

Proof of Lemma S6. Referring to Figure S3, we can compute

$$\mathbb{E}(L_A(ab|cd)) = \Bigg(\int_0^{T_1}\int_0^{T_2} e^{-x}e^{-y}(x\mu_1 + T_A\mu_A)\,dy\,dx \qquad \text{(scenario (a))}$$
$$+ e^{-T_2}\int_0^{T_1}\int_0^{\infty} e^{-x}3e^{-3y}\frac{1}{3}(x\mu_1 + T_A\mu_A)\,dy\,dx \qquad \text{(scenario (b))}$$
$$+ e^{-T_2}\int_0^{T_1}\int_0^{\infty} e^{-x}3e^{-3y}\frac{2}{3}(x\mu_1 + T_A\mu_A)\,dy\,dx \qquad \text{(scenario (c))}$$
$$+ 2e^{-T_1}\int_0^{\infty}\int_0^{T_2} 3e^{-3x}e^{-y}\frac{1}{3}(x\mu_3 + T_1\mu_1 + T_A\mu_A)\,dy\,dx \qquad \text{(scenario (d), (e))}$$
$$+ e^{-T_1}\int_0^{\infty}\int_0^{T_2} 3e^{-3x}e^{-y}\frac{1}{3}((x+2)\mu_3 + T_1\mu_1 + T_A\mu_A)\,dy\,dx \qquad \text{(scenario (e))}$$
$$+ 2e^{-T_1}e^{-T_2}\int_0^{\infty}\int_x^{\infty} 6e^{-6x}3e^{-3(y-x)}\frac{1}{6\times 3}(y\mu_3 + T_1\mu_1 + T_A\mu_A)\,dy\,dx \qquad \text{(scenario (f), (g))}$$
$$+ e^{-T_1}e^{-T_2}\int_0^{\infty}\int_x^{\infty} 6e^{-6x}3e^{-3(y-x)}\frac{1}{6\times 3}((y+2)\mu_3 + T_1\mu_1 + T_A\mu_A)\,dy\,dx \qquad \text{(scenario (g))}$$
$$+3e^{-T_1}e^{-T_2}\int_0^{\infty}\int_x^{\infty} 6e^{-6x}3e^{-3(y-x)}\frac{1}{6\times 3}(x\mu_3 + T_1\mu_1 + T_A\mu_A)\,dy\,dx\Bigg) \Big/ \left(1 - \frac{2}{3}e^{-(T_1+T_2)}\right) \qquad \text{(scenario (f), (g))}$$
$$= \frac{e^{-(T_1+T_2)}(-6T_1\mu_1 - 7\mu_3) + 9((1-e^{-T_1})\mu_1 + \mu_3 e^{-T_1})}{9 - 6e^{-(T_1+T_2)}} + \mu_A T_A \tag{S32}$$

Similarly, referring to Figure S4, we can compute

$$\mathbb{E}(L_A(ad|bc)) = \mathbb{E}(L_A(ac|bd)) =$$
$$\Bigg(2e^{-T_1}e^{-T_2}\int_0^{\infty}\int_x^{\infty} 6e^{-6x}3e^{-3(y-x)}\frac{1}{6\times 3}(y\mu_3 + T_1\mu_1 + T_A\mu_A)\,dy\,dx \qquad \text{(scenario (a), (b))}$$
$$+ e^{-T_1}e^{-T_2}\int_0^{\infty}\int_x^{\infty} 6e^{-6x}3e^{-3(y-x)}\frac{1}{6\times 3}((y+2)\mu_3 + T_1\mu_1 + T_A\mu_A)\,dy\,dx \qquad \text{(scenario (b))}$$
$$+3e^{-T_1}e^{-T_2}\int_0^{\infty}\int_x^{\infty} 6e^{-6x}3e^{-3(y-x)}\frac{1}{6\times 3}(x\mu_3 + T_1\mu_1 + T_A\mu_A)\,dy\,dx\Bigg) \Big/ \left(\frac{1}{3}e^{-(T_1+T_2)}\right) \qquad \text{(scenario (a), (b))}$$
$$= T_1\mu_1 + \frac{2}{3}\mu_3 + \mu_A T_A \tag{S33}$$

□

Using these lemmas, we now prove Theorem S1.

Proof of Theorem S1. Subtracting Eq. S31 from Eq. S30, we get

$$\Delta_I = \mathbb{E}(L_I(ab|cd)) - \mathbb{E}(L_I(ac|bd)) = \mathbb{E}(L_I(ab|cd)) - \mathbb{E}(L_I(ad|bc)) \tag{S34}$$
$$= \frac{3(e^{-T_1}(\mu_1 - \mu_3) + \mu_3 e^{-(T_1+T_2)} + e^{-T_2}(\mu_2 - \mu_3) + (T_1\mu_1 + T_2\mu_2 - \mu_1 - \mu_2 + \mu_3))}{3 - 2e^{-(T_1+T_2)}} \tag{S35}$$

Subtracting Eq. S33 from Eq. S32, we get

$$\Delta_A = \mathbb{E}(L_A(ab|cd)) - \mathbb{E}(L_A(ad|bc) = \mathbb{E}(L_A(ab|cd)) - \mathbb{E}(L_A(ac|bd))) \tag{S36}$$
$$= \frac{-\mu_3 e^{-(T_1+T_2)} + 3\mu_1(1 - e^{-T_1} - T_1) + \mu_3(-2 + 3e^{-T_1})}{-2e^{-(T_1+T_2)} + 3} \tag{S37}$$

□

Simplifications for balanced equations. Similar to the simplifications for the unbalanced tree equations (explained in the main text), we simplify equations of Theorem S1 by computing their limit as $T_2 \to 0$ or $\mu_3 \to \mu_1$.

To compute the length of the internal branch, i.e. $t_1 + t_2$, we simplify Equation (S24) by computing its limit as $T_2 \to 0$ (note that by symmetry, $T_1 \to 0$ can also be used and will lead to the same final formula).

$$\lim_{T_2 \to 0} \Delta_I = \frac{3\mu_1(e^{-T_1} - 1 + T_1)}{3 - 2e^{-T_1}} \tag{S38}$$

which is exactly the same as Equation (5) for unbalanced trees. Note that by eq. (S27), we have $\bar{L}'_I = \mu_3$, therefore, further assuming $\mu_1 = \mu_3$ allows us to estimate μ_1 as the average length of the internal branch in non-matching gene trees. We replace Δ_I with the observed difference between average internal branch lengths in matching and non-matching gene trees in Equation (S38), leading to the same equation as eq. (6) for unbalanced trees. The solution to this equation is based on the Lambert W function (see main text), which we approximate using a Taylor approximation as in Equation (7), leading to the following formula:

$$\hat{T_1}\hat{\mu_1} = \bar{L}'_I \left(\frac{1}{2}\bar{\delta} + \frac{1}{6}\sqrt{3\bar{\delta}(3\bar{\delta} + 4)} \right) \tag{S39}$$

where $\bar{\delta} = \frac{\bar{\Delta}_I}{\bar{L}'_I}$. Note that since we initially assumed $T_2 \to 0$, the length of the internal branch in the limit only depends on $\hat{T_1}\hat{\mu_1}$, and we use Equation (S39) as an estimate of $t_1 + t_2$ (the same estimator can be derived using $T_1 \to 0$).

For the terminal branch of A, assuming $\mu_3 \to \mu_1$, we can simplify Equations (S25) and (S29), as follows:

$$\lim_{\mu_3 \to \mu_1} \Delta_A = \frac{\mu_1 \left(-e^{-(T_1+T_2)} + 1 - 3T_1 \right)}{-2e^{-(T_1+T_2)} + 3} \tag{S40}$$

and

$$\lim_{\mu_3 \to \mu_1} L'_A = T_1\mu_1 + \frac{2}{3}\mu_1 + \mu_A T_A \tag{S41}$$

We replace the expected value $\lim_{\mu_3 \to \mu_1} L'_A$ in Equation (S41) with the observed mean difference $\bar{\Delta}_A$ and $\lim_{\mu_3 \to \mu_1} L'_A$ with the observed mean $\bar{L}'_A$. Solving for $\mu_A T_A$ gives the following estimate for t_A:

$$\hat{t_A} = \bar{L}'_A - \frac{2}{3}\mu_1 - \frac{1}{3}\left(\mu_1 \left(1 - e^{-(T_1+T_2)} \right) - \bar{\Delta}_A \left(3 - 2e^{-(T_1+T_2)} \right) \right) \tag{S42}$$

Note that due to symmetry, all nodes in a balanced quartet are part of a cherry, and therefore the same equations and simplifications can be used for all terminal branches, only replacing the appropriate Ts and μs. In particular, for C and D, we use a different assumption $\mu_3 \to \mu_2$, so that the final equation depends on T_2 and μ_2, i.e. parameteres of the branch above the cherry. Tables S1 to S3 summarize the expected lengths, simplified formulas and the final branch length estimators for both unbalanced and balanced trees.

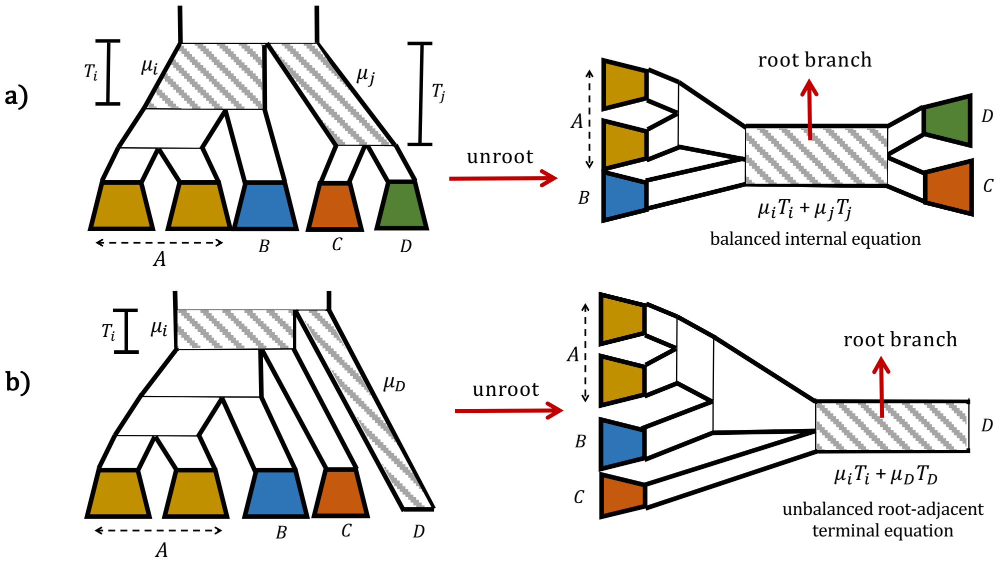

Figure S5: **Root branch calculation based on balanced or unbalanced quartets.** The root branch in the unrooted species tree corresponds to two branches in the rooted tree (the highlighted branches). Our equations calculate the *sum* of the two root-adjacent branches, that correspond to a single branch in the unrooted species tree. Hence, the length of the individual parts of this branch, and therefore the exact position of the root, is not inferred in our approach. a) When none of the children of the root is a leaf, then the length of the root branch can be computed from balanced quartets, using the internal branch equation. b) When one child of the root is a leaf, the root branch length can be calculated using the equations for the root-adjacent terminal branch (referred to as terminal D in the proofs) from unbalanced quartets.

Table S1: Summary of formulas for expected branch lengths in matching and non-matching gene trees.

Unbalanced

Parameter	Formula	Derivation
L_I	$\frac{(e^{-3T_2}+3e^{-T_2}-6e^{T_1-T_2})(\mu_2-\mu_3)+6(1-e^{T_1}+T_1e^{T_1})\mu_1}{2(3e^{T_1}-2)}+\mu_2$	Lemma S1 (Eq. S9)
L'_I	$\mu_2+\frac{1}{2}(\mu_2-\mu_3)\left(e^{-3T_2}-3e^{-T_2}\right)$	Lemma S1 (Eq. S10)
Δ_I	$\frac{3(e^{-T_2}-e^{-3T_2})(1-e^{-T_1})(\mu_2-\mu_3)+6\mu_1(e^{-T_1}-1+T_1)}{2(3-2e^{-T_1})}$	Theorem 1 (Eq. S18)
L_A	$\frac{6T_1\mu_1+3\mu_1-\mu_2+e^{-3T_2}(\mu_2-2\mu_3)}{6-9e^{T_1}}+\mu_1+\mu_AT_A$	Lemma S2 (Eq. S11)
L'_A	$\frac{1}{12}(10\mu_2-9e^{-T_2}(\mu_2-\mu_3)-3^{-3T_2}(\mu_2+\mu_3))+T_1\mu_1+T_A\mu_A$	Lemma S2 (Eq. S19)
Δ_A	$\frac{\left(4\mu_2-6\mu_1-(3e^{-T_2}+e^{-3T_2})(\mu_2-\mu_3)+e^{T_1}\left(\frac{1}{2}(\mu_2+\mu_3)e^{-3T_2}+\frac{9}{2}e^{-T_2}(\mu_2-\mu_3)-6T_1\mu_1+6\mu_1-5\mu_2\right)\right)}{2(-2+3e^{T_1})}$	Theorem 1 (Eq. S21)
L_C	$-e^{-T_2}(\mu_2-\mu_3)+\mu_2+\mu_CT_C+\frac{2\mu_2-\left(3e^{-T_2}-e^{-3T_2}\right)(\mu_2-\mu_3)-4\mu_3e^{-3T_2}}{6(3e^{T_1}-2)}$	Lemma S3 (Eq. S14)
L'_C	$\frac{1}{3}\mu_2(1+e^{-3T_2})+\mu_CT_C$	Lemma S3 (Eq. S15)
Δ_C	$\frac{(2-e^{-T_1})\left(\left(e^{-3T_2}+2\right)\mu_2-3e^{-T_2}(\mu_2-\mu_3)\right)+\mu_3e^{-3T_2}(e^{-T_1}-4)}{2(3-2e^{-T_1})}$	Theorem 1 (Eq. S22)
L_D	$e^{-T_2}(\mu_2-\mu_3)-\mu_2+2\mu_3+T_2\mu_2+\mu_DT_D+\frac{-2\mu_2+\left(3e^{-T_2}-e^{-3T_2}\right)(\mu_2-\mu_3)}{6(3e^{T_1}-2)}$	Lemma S4 (Eq. S16)
L'_D	$\left(\frac{3}{2}e^{-T_2}-\frac{1}{6}e^{-3T_2}\right)(\mu_2-\mu_3)-\frac{4}{3}\mu_2+2\mu_3+T_2\mu_2+\mu_DT_D$	Lemma S4 (Eq. S17)
Δ_D	$\frac{(1-e^{-T_1})(2\mu_2-(3e^{-T_2}-e^{-3T_2})(\mu_2-\mu_3))}{2(3-2e^{-T_1})}$	Theorem 1 (Eq. S23)

Balanced

L_I	$\frac{3e^{-T_1}(\mu_1-\mu_3)+\mu_3e^{-(T_1+T_2)}+3e^{-T_2}(\mu_2-\mu_3)+3(T_1\mu_1+T_2\mu_2-\mu_1-\mu_2+2\mu_3)}{3-2e^{-(T_1+T_2)}}$	Lemma S5 (Eq. S30)
L'_I	μ_3	Lemma S5 (Eq. S31)
Δ_I	$\frac{3(e^{-T_1}(\mu_1-\mu_3)+\mu_3e^{-(T_1+T_2)}+e^{-T_2}(\mu_2-\mu_3)+(T_1\mu_1+T_2\mu_2-\mu_1-\mu_2+\mu_3))}{3-2e^{-(T_1+T_2)}}$	Theorem S1 (Eq. S35)
L_A	$\frac{e^{-(T_1+T_2)}(-6T_1\mu_1-7\mu_3)+9((1-e^{-T_1})\mu_1+\mu_3e^{-T_1})}{9-6e^{-(T_1+T_2)}}+\mu_AT_A$	Lemma S6 (Eq. S32)
L'_A	$T_1\mu_1+\frac{2}{3}\mu_3+\mu_AT_A$	Lemma S6 (Eq. S33)
Δ_A	$\frac{-\mu_3e^{-(T_1+T_2)}+3\mu_1(1-e^{-(T_1)}-T_1)+\mu_3(-2+3e^{-(T_1)})}{-2e^{-(T_1+T_2)}+3}$	Theorem S1 (Eq. S37)

Table S2: Summary of simplifying assumptions and the corresponding simplified formulas for expected branch lengths in matching and non-matching gene trees.

Unbalanced

Parameter	Simplified formula	Simplifying assumption
L_I	$\lim_{\mu_3\to\mu_2} L_I = \frac{3\mu_1(e^{-T_1}-1+T_1)}{3-2e^{-T_1}} + \mu_2$	$\mu_3 \to \mu_2$
L'_I	$\lim_{\mu_3\to\mu_2} L'_I = \mu_2$	$\mu_3 \to \mu_2$
Δ_I	$\lim_{\mu_3\to\mu_2} \Delta_I = \frac{3\mu_1(e^{-T_1}-1+T_1)}{3-2e^{-T_1}}$	$\mu_3 \to \mu_2$
L_A	$\lim_{T_2\to\infty} L_A = \frac{6T_1\mu_1+3\mu_1-\mu_2}{6-9e^{T_1}} + \mu_1 + \mu_A T_A$	$T_2 \to \infty$
L'_A	$\lim_{T_2\to\infty} L'_A = \frac{5}{6}\mu_2 + T_1\mu_1 + T_A\mu_A$	$T_2 \to \infty$
Δ_A	$\lim_{T_2\to\infty} \Delta_A = \frac{-6\mu_1\left(e^{-T_1}-1+T_1\right)-(5-4e^{-T_1})\mu_2}{6-4e^{-T_1}}$	$T_2 \to \infty$
L_C	$\lim_{T_2\to\infty} L_C = \mu_2 + \mu_C T_C + \frac{\mu_2}{3(3e^{T_1}-2)}$	$T_2 \to \infty$
L'_C	$\lim_{T_2\to\infty} L'_C = \frac{1}{3}\mu_2 + \mu_C T_C$	$T_2 \to \infty$
Δ_C	$\lim_{T_2\to\infty} \Delta_C = \frac{(2-e^{-T_1})\mu_2}{(3-2e^{-T_1})}$	$T_2 \to \infty$
L_D	$\lim_{\mu_3\to\mu_2} L_D = \mu_2 + T_2\mu_2 + \mu_D T_D - \frac{\mu_2}{3(3e^{T_1}-2)}$	$\mu_3 \to \mu_2$
L'_D	$\lim_{\mu_3\to\mu_2} L'_D = \frac{2}{3}\mu_2 + T_2\mu_2 + \mu_D T_D$	$\mu_3 \to \mu_2$
Δ_D	$\lim_{\mu_3\to\mu_2} \Delta_D = \frac{(1-e^{-T_1})\mu_2}{3-2e^{-T_1}}$	$\mu_3 \to \mu_2$

Balanced

L_I	$\lim_{T_2\to 0} L_I = \frac{3\mu_1(e^{-T_1}-1+T_1)}{3-2e^{-T_1}} + \mu_3$	$T_2 \to 0$
L'_I	μ_3	—
Δ_I	$\lim_{T_2\to 0} \Delta_I = \frac{3\mu_1(e^{-T_1}-1+T_1)}{3-2e^{-T_1}}$	$T_2 \to 0$
L_A	$\lim_{\mu_3\to\mu_1} L_A = \mu_1 + \frac{\mu_1(1+6T_1)}{6-9e^{(T_1+T_2)}} + \mu_A T_A$	$\mu_3 \to \mu_1$
L'_A	$\lim_{\mu_3\to\mu_1} L'_A = T_1\mu_1 + \frac{2}{3}\mu_1 + \mu_A T_A$	$\mu_3 \to \mu_1$
Δ_A	$\lim_{\mu_3\to\mu_1} \Delta_A = \frac{\mu_1\left(-e^{-(T_1+T_2)}+1-3T_1\right)}{-2e^{-(T_1+T_2)}+3}$	$\mu_3 \to \mu_1$

Table S3: Summary of formulas for estimating unbalanced or balanced species tree branch lengths in SU. Note that for an unbalanced species tree of Figure 1, the internal branch has length t_1 in SU, but for the balanced species tree it has length $t_1 + t_2$, as it is the root branch. For each branch X, $\bar{L}_X$ and $\bar{L}'_X$ refer to the *observed* mean branch length in matching or non-matching gene trees. For both balanced and unbalanced internal branch, $\bar{\delta}$ is defined as $\bar{\delta} = \frac{\bar{\Delta}_I}{\bar{L}'_I}$. The length of the internal branch does not depend on CU lengths, but the equations for the terminal branches use the CU length of the adjacent internal branch (i.e. T_1 for unbalanced tree and $T_1 + T_2$ for balanced tree) which is computed using the approach from Sayyari and Mirarab (2016) inside CASTLES.

Unbalanced

Parameter	Estimation formula	Simplifying assumption(s)
t_1	$\hat{t_1} = \bar{L}'_I\left(\frac{1}{2}\bar{\delta} + \frac{1}{6}\sqrt{3\bar{\delta}(3\bar{\delta}+4)}\right)$	$\mu_3 \to \mu_2; \mu_1 \to \mu_2$
t_A	$\hat{t_A} = \bar{L}'_A + \frac{\mu_1(e^{-T_1}-1+T_1)+\bar{\Delta}_A(1-2/3e^{-T_1})}{1-4/5e^{-T_1}} - T_1\mu_1$	$T_2 \to \infty$
t_B	$\hat{t_B} = \bar{L}'_B + \frac{\mu_1(e^{-T_1}-1+T_1)+\bar{\Delta}_B(1-2/3e^{-T_1})}{1-4/5e^{-T_1}} - T_1\mu_1$	$T_2 \to \infty$
t_C	$\hat{t_C} = \bar{L}'_C - \frac{1}{3}(2 - \frac{1}{2-e^{-T_1}})\bar{\Delta}_C$	$T_2 \to \infty$
$t_2 + t_D$	$\hat{t_2} + \hat{t_D} = \bar{L}'_D - \frac{2}{3}(2 + \frac{1}{1-e^{-T_1}})\bar{\Delta}_D$	$\mu_3 \to \mu_2$

Balanced

$t_1 + t_2$	$\hat{t_1} + \hat{t_2} = \bar{L}'_I\left(\frac{1}{2}\bar{\delta} + \frac{1}{6}\sqrt{3\bar{\delta}(3\bar{\delta}+4)}\right)$	$T_2 \to 0; \mu_1 \to \mu_3$
t_A	$\hat{t_A} = \bar{L}'_A - \frac{2}{3}\mu_1 - \frac{1}{3}\left(\mu_1\left(1-e^{-(T_1+T_2)}\right) - \bar{\Delta}_A\left(3-2e^{-(T_1+T_2)}\right)\right)$	$\mu_3 \to \mu_1$
t_B	$\hat{t_B} = \bar{L}'_B - \frac{2}{3}\mu_1 - \frac{1}{3}\left(\mu_1\left(1-e^{-(T_1+T_2)}\right) - \bar{\Delta}_B\left(3-2e^{-(T_1+T_2)}\right)\right)$	$\mu_3 \to \mu_1$
t_C	$\hat{t_C} = \bar{L}'_C - \frac{2}{3}\mu_2 - \frac{1}{3}\left(\mu_2\left(1-e^{-(T_1+T_2)}\right) - \bar{\Delta}_C\left(3-2e^{-(T_1+T_2)}\right)\right)$	$\mu_3 \to \mu_2$
t_D	$\hat{t_D} = \bar{L}'_D - \frac{2}{3}\mu_2 - \frac{1}{3}\left(\mu_2\left(1-e^{-(T_1+T_2)}\right) - \bar{\Delta}_D\left(3-2e^{-(T_1+T_2)}\right)\right)$	$\mu_3 \to \mu_2$

S2 Dynamic Programming Algorithm

In this section, we will provide an $O(n^2)$ large tree algorithm for computing branch lengths for all internal and terminal branches. For conciseness, we use A, B, C, D to denote sets of taxa and use a, b, c, d to denote individual taxa. To compute all branch lengths, it is sufficient to compute the following counters for a set of ordered leafset quadripartitions, in which each leafset quadripartition (A, B, C, D) – up to permutations – corresponds to an internal branch:

- $n(A, B; C, D)$: the number of quartet and gene tree combinations $(a, b, c, d, G) \in A \times B \times C \times D \times \mathcal{G}$ such that $G \upharpoonright \{a, b, c, d\}$ has topology $ab|cd$.
- $x(A, B; C, D)$: the total internal branch lengths of quartet trees in the form of $G \upharpoonright \{a, b, c, d\}$ with topology $ab|cd$, where $(a, b, c, d, G) \in A \times B \times C \times D \times \mathcal{G}$.
- $a(A; B; C, D)$: the total length of the terminal branches leading to A in quartet trees in the form of $G \upharpoonright \{a, b, c, d\}$ with topology $ab|cd$, where $(a, b, c, d, G) \in A \times B \times C \times D \times \mathcal{G}$.

All three counters for each quadripartition (A, B, C, D) can be computed in a single post-order traversal of the gene tree nodes in $O(n)$ using Algorithm S1. Therefore, computing counters for all $O(n)$ quadripartitions has time complexity $O(n^2)$. Notice that Algorithm S1 assumes that all input gene trees are fully resolved. Otherwise, input gene trees should be arbitrarily resolved by adding ghost branches of zero lengths (not counted towards $n(A, B; C, D)$).

Algorithm S1 Large tree algorithm. The input is a set of gene trees $\mathcal{G}$ and an ordered quadripartition of its leafset (A, B, C, D), and the output are $n(A, B; C, D)$, $x(A, B; C, D)$, and $a(A; B; C, D)$. For each node u we keep a list of counters $C^{\cdot}_{\cdot}(u)$ described in Table S4.

procedure UpdateLeafCounters(u, A, B, C, D)
    Set all counters $C^{\cdot}_{\cdot}(u)$ to 0
    if u corresponds to a taxon in A **then**
        $C^1_A(u) \leftarrow 1$
        $C^a_A(u) \leftarrow$ the parental branch length of u
    else if u corresponds to a taxon in B **then**
        $C^1_B(u) \leftarrow 1$
        $C^b_B(u) \leftarrow$ the parental branch length of u
    else if u corresponds to a taxon in C **then**
        $C^1_C(u) \leftarrow 1$
        $C^c_C(u) \leftarrow$ the parental branch length of u
    else if u corresponds to a taxon in D **then**
        $C^1_D(u) \leftarrow 1$
        $C^d_D(u) \leftarrow$ the parental branch length of u
    end if
end procedure
procedure LargeTreeAlgorithm($\mathcal{G}$, A, B, C, D)
    Set $n(A, B; C, D)$, $x(A, B; C, D)$, $a(A; B; C, D)$ to 0
    for each gene $G \in \mathcal{G}$ **do**
        for $u \in$ post order traverse of internal nodes of G **do**
            if u is a leaf node **then**
                UpdateLeafCounters(u, A, B, C, D)
            else
                Update all counters $C^{\cdot}_{\cdot}(u)$ using the recursive formula in Table S4
                $n(A, B; C, D) \leftarrow n(A, B; C, D) + C^1_{AB|CD}(w)$
                $x(A, B; C, D) \leftarrow x(A, B; C, D) + C^x_{AB|CD}(w)$
                $a(A, B; C, D) \leftarrow a(A, B; C, D) + C^a_{AB|CD}(w)$
            end if
        end for
    end for
end procedure

Table S4: We define several counters for every node w, each of which computes $\sum_{e \in S(w)} f(e)$ for some S and f. We define several notations: let p denote the parent node of w and u, v denote the child nodes of w; let $\mathcal{L}(w)$ denote the set of leaves under w; let $\mathcal{D}(\cdot, \cdot)$ denote the distance of two nodes; let $\mathcal{M}(\cdot, \cdot)$ denote the most recent common ancestor of two nodes; let $\mathcal{H}(\cdot, \cdot)$ be the 0/1 indicator of whether all branches on the path between the two nodes are ghost branches. Superscripts $-$ and $+$ signify ghost branches and all branches, respectively. Note that A and B are interchangeable in the names; e.g., $C^{1+}_{BA}(w)$ is defined similarly to $C^{1+}_{AB}(w)$. Table continues to the next page. See Figures S6 and S7 for illustrations of recursive formulas.

Counter	Set $S(w)$	Function $f(e)$	Recursive formula	Similarly defined
$C^1_A(w)$	$a : a \in A \cap \mathcal{L}(w)$	1	$C^1_A(u) + C^1_A(v)$	$C^1_B(w)$ ...
$C^a_A(w)$	$a : a \in A \cap \mathcal{L}(w)$	$\mathcal{D}(a, p)$	$C^a_A(u) + C^a_A(v) + \mathcal{D}(w, p)C^1_A(w)$	$C^b_B(w)$ ...
$C^{1+}_{AB}(w)$	$(a, b) : a \in A \cap \mathcal{L}(w), b \in B \cap \mathcal{L}(w)$	1	$C^{1+}_{AB}(u) + C^{1+}_{AB}(v) + C^1_A(u)C^1_B(v) + C^1_B(u)C^1_A(v)$	$C^{1+}_{AC}(w)$ ...
$C^{1-}_{AB}(w)$	$(a, b) : a \in A \cap \mathcal{L}(w), b \in B \cap \mathcal{L}(w), \mathcal{H}(\mathcal{M}(a, b), p) = 1$	1	$(C^{1-}_{AB}(u) + C^{1-}_{AB}(v) + C^1_A(u)C^1_B(v) + C^1_B(u)C^1_A(v))\mathcal{H}(w, p)$	$C^{1-}_{AC}(w)$ ...
$C^1_{AB}(w)$	$(a, b) : a \in A \cap \mathcal{L}(w), b \in B \cap \mathcal{L}(w), \mathcal{H}(\mathcal{M}(a, b), p) \neq 1$	1	$C^{1+}_{AB}(w) - C^{1-}_{AB}(w)$	$C^1_{AC}(w)$ ...
$C^{a+}_{AB}(w)$	$(a, b) : a \in A \cap \mathcal{L}(w), b \in B \cap \mathcal{L}(w)$	$\mathcal{D}(a, \mathcal{M}(a, b))$	$C^{a+}_{AB}(u) + C^{a+}_{AB}(v) + C^a_A(u)C^1_B(v) + C^1_B(u)C^a_A(v)$	$C^{b+}_{AB}(w)$, $C^{a+}_{AC}(w)$ ...
$C^{a-}_{AB}(w)$	$(a, b) : a \in A \cap \mathcal{L}(w), b \in B \cap \mathcal{L}(w), \mathcal{H}(\mathcal{M}(a, b), p) = 1$	$\mathcal{D}(a, \mathcal{M}(a, b))$	$(C^{a-}_{AB}(u) + C^{a-}_{AB}(v) + C^a_A(u)C^1_B(v) + C^1_B(u)C^a_A(v))\mathcal{H}(w, p)$	$C^{b-}_{AB}(w)$, $C^{a-}_{AC}(w)$ ...
$C^a_{AB}(w)$	$(a, b) : a \in A \cap \mathcal{L}(w), b \in B \cap \mathcal{L}(w), \mathcal{H}(\mathcal{M}(a, b), p) \neq 1$	$\mathcal{D}(a, \mathcal{M}(a, b))$	$C^{a+}_{AB}(w) - C^{a-}_{AB}(w)$	$C^b_{AB}(w)$, $C^a_{AC}(w)$ ...
$C^{x+}_{AB}(w)$	$(a, b) : a \in A \cap \mathcal{L}(w), b \in B \cap \mathcal{L}(w)$	$\mathcal{D}(\mathcal{M}(a, b), p)$	$C^{x+}_{AB}(u) + C^{x+}_{AB}(v) + \mathcal{D}(w, p)C^{1+}_{AB}(w)$	$C^{x+}_{AC}(w)$ ...
$C^{x-}_{AB}(w)$	$(a, b) : a \in A \cap \mathcal{L}(w), b \in B \cap \mathcal{L}(w), \mathcal{H}(\mathcal{M}(a, b), p) = 1$	$\mathcal{D}(\mathcal{M}(a, b), p)$	$(C^{x-}_{AB}(u) + C^{x-}_{AB}(v) + \mathcal{D}(w, p)C^{1-}_{AB}(w))\mathcal{H}(w, p)$	$C^{x-}_{AC}(w)$ ...
$C^x_{AB}(w)$	$(a, b) : a \in A \cap \mathcal{L}(w), b \in B \cap \mathcal{L}(w), \mathcal{H}(\mathcal{M}(a, b), p) \neq 1$	$\mathcal{D}(\mathcal{M}(a, b), p)$	$C^{x+}_{AB}(w) - C^{x-}_{AB}(w)$	$C^x_{AC}(w)$ ...
$C^1_{C\|AB}(w)$	$(a, b, c) : a \in A \cap \mathcal{L}(w), b \in B \cap \mathcal{L}(w), c \in C \cap \mathcal{L}(w), \mathcal{L}(\mathcal{M}(a, b)) \subsetneq \mathcal{L}(\mathcal{M}(a, c)), \mathcal{H}(\mathcal{M}(a, b), \mathcal{M}(a, c)) \neq 1$	1	$C^1_{C\|AB}(u) + C^1_{C\|AB}(v) + C^1_{AB}(u)C^1_C(v) + C^1_C(u)C^1_{AB}(v)$	$C^1_{A\|BC}(w)$ ...
$C^a_{C\|AB}(w)$	same as above	$\mathcal{D}(a, \mathcal{M}(a, b))$	$C^a_{C\|AB}(u) + C^a_{C\|AB}(v) + C^a_{AB}(u)C^1_C(v) + C^1_C(u)C^a_{AB}(v)$	$C^b_{C\|AB}(w)$, $C^b_{A\|BC}(w)$ ...
$C^c_{C\|AB}(w)$	same as above	$\mathcal{D}(c, \mathcal{M}(a, c))$	$C^c_{C\|AB}(u) + C^c_{C\|AB}(v) + C^1_{AB}(u)C^c_C(v) + C^c_C(u)C^1_{AB}(v)$	$C^a_{A\|BC}(w)$ ...
$C^d_{C\|AB}(w)$	same as above	$\mathcal{D}(\mathcal{M}(a, c), p)$	$C^d_{C\|AB}(u) + C^d_{C\|AB}(v) + \mathcal{D}(w, p)C^1_{C\|AB}(w)$	$C^d_{A\|BC}(w)$ ...
$C^x_{C\|AB}(w)$	same as above	$\mathcal{D}(\mathcal{M}(a, b), \mathcal{M}(a, c))$	$C^x_{C\|AB}(u) + C^x_{C\|AB}(v) + C^x_{AB}(u)C^1_C(v) + C^1_C(u)C^x_{AB}(v)$	$C^x_{A\|BC}(w)$ ...

Here, we omit the column $S(w)$. For all $C^{\cdot}_{AB|CD}(w)$ show below, $S(w)$ is the set of quartets a, b, c, d, whose MRCA is w, form a tree with topology $ab|cd$ and are elements of sets A, B, C, D, respectively.

Counter	Function $f(e)$	Recursive formula	Similarly defined
$C^{1}_{AB\|CD}(w)$	1	$C^{1}_{C\|AB}(u)C^{1}_{D}(v) + C^{1}_{D}(u)C^{1}_{C\|AB}(v) + C^{1}_{D\|AB}(u)C^{1}_{C}(v) + C^{1}_{C}(u)C^{1}_{D\|AB}(v) + C^{1}_{B\|CD}(u)C^{1}_{A}(v) + C^{1}_{A}(u)C^{1}_{B\|CD}(v) + C^{1}_{A\|CD}(u)C^{1}_{B}(v) + C^{1}_{B}(u)C^{1}_{A\|CD}(v) + C^{1+}_{AB}(u)C^{1+}_{CD}(v) - C^{1-}_{AB}(u)C^{1-}_{CD}(v) + C^{1+}_{CD}(u)C^{1+}_{AB}(v) - C^{1-}_{CD}(u)C^{1-}_{AB}(v)$	$C^{1}_{AB\|CD}(w)$, $C^{1}_{AC\|BD}(w)$
$C^{a}_{AB\|CD}(w)$	$\mathcal{D}(a, \mathcal{M}(a, b))$	$C^{a}_{C\|AB}(u)C^{1}_{D}(v) + C^{1}_{D}(u)C^{a}_{C\|AB}(v) + C^{a}_{D\|AB}(u)C^{1}_{C}(v) + C^{1}_{C}(u)C^{a}_{D\|AB}(v) + C^{a}_{B\|CD}(u)C^{1}_{A}(v) + C^{1}_{B\|CD}(u)C^{a}_{A}(v) + C^{a}_{A}(u)C^{1}_{B\|CD}(v) + C^{1}_{A}(u)C^{a}_{B\|CD}(v) + C^{a}_{A\|CD}(u)C^{1}_{B}(v) + C^{1}_{B}(u)C^{a}_{A\|CD}(v) + C^{a+}_{AB}(u)C^{1+}_{CD}(v) - C^{a-}_{AB}(u)C^{1-}_{CD}(v) + C^{1+}_{CD}(u)C^{a+}_{AB}(v) - C^{1-}_{CD}(u)C^{a-}_{AB}(v)$	$C^{b}_{AB\|CD}(w)$, $C^{a}_{AC\|BD}(w)$ ...
$C^{x}_{AB\|CD}(w)$	$\mathcal{D}(\mathcal{M}(a, b), \mathcal{M}(c, d))$	$C^{x}_{C\|AB}(u)C^{1}_{D}(v) + C^{1}_{D}(u)C^{x}_{C\|AB}(v) + C^{x}_{D\|AB}(u)C^{1}_{C}(v) + C^{1}_{C}(u)C^{x}_{D\|AB}(v) + C^{x}_{B\|CD}(u)C^{1}_{A}(v) + C^{1}_{A}(u)C^{x}_{B\|CD}(v) + C^{x}_{A\|CD}(u)C^{1}_{B}(v) + C^{1}_{B}(u)C^{x}_{A\|CD}(v) + C^{x+}_{AB}(u)C^{1+}_{CD}(v) - C^{x-}_{AB}(u)C^{1-}_{CD}(v) + C^{1+}_{AB}(u)C^{x+}_{CD}(v) - C^{1-}_{AB}(u)C^{x-}_{CD}(v) + C^{x+}_{CD}(u)C^{1+}_{AB}(v) - C^{x-}_{CD}(u)C^{1-}_{AB}(v) + C^{1+}_{CD}(u)C^{x+}_{AB}(v) - C^{1-}_{CD}(u)C^{x-}_{AB}(v)$	$C^{x}_{AC\|BD}(w)$, $C^{x}_{AD\|BC}(w)$

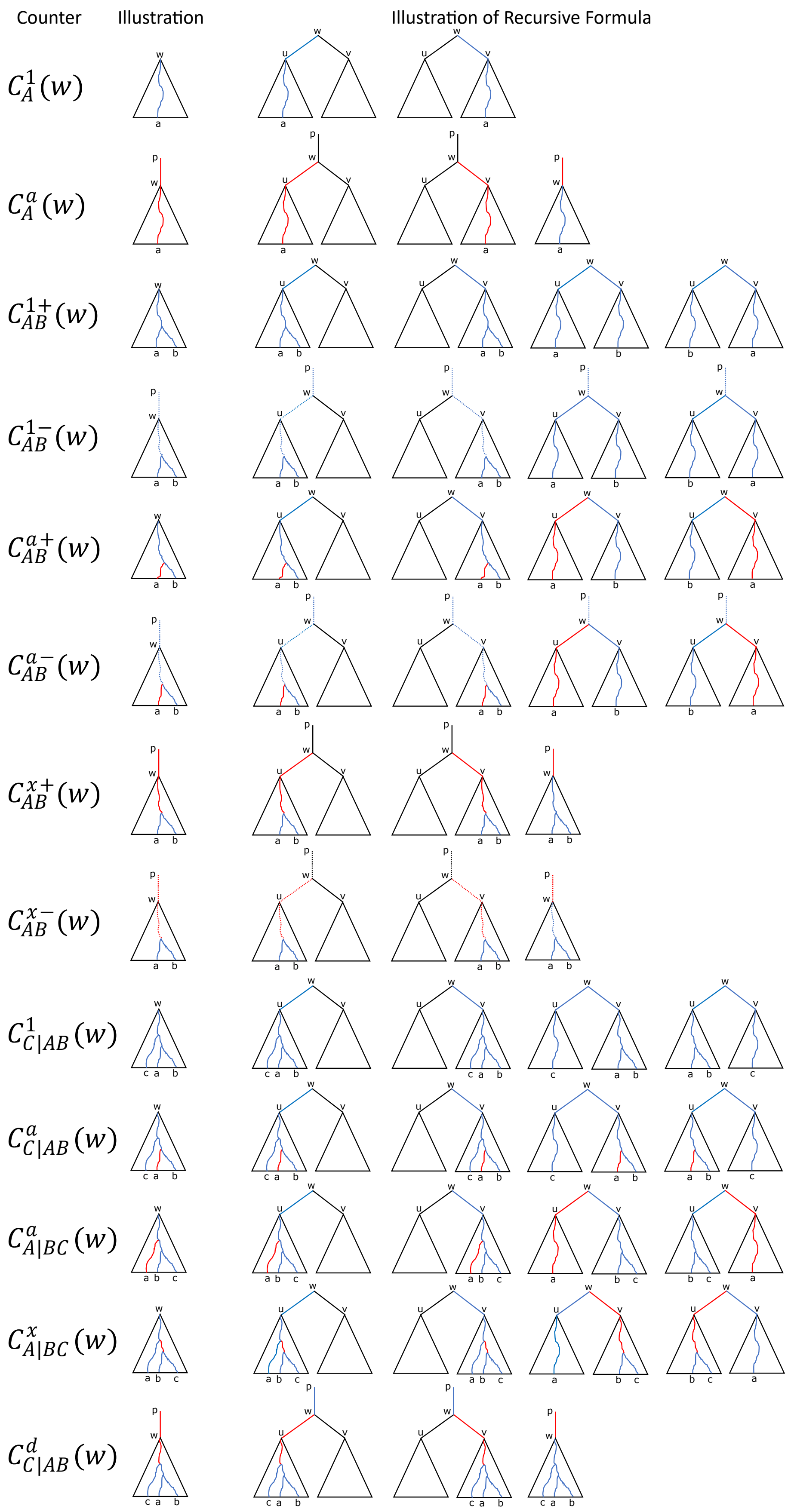

Figure S6: Illustration of counters and their recursive formulas. Branches colored red are counted by lengths, and dotted branches must be ghost branches.

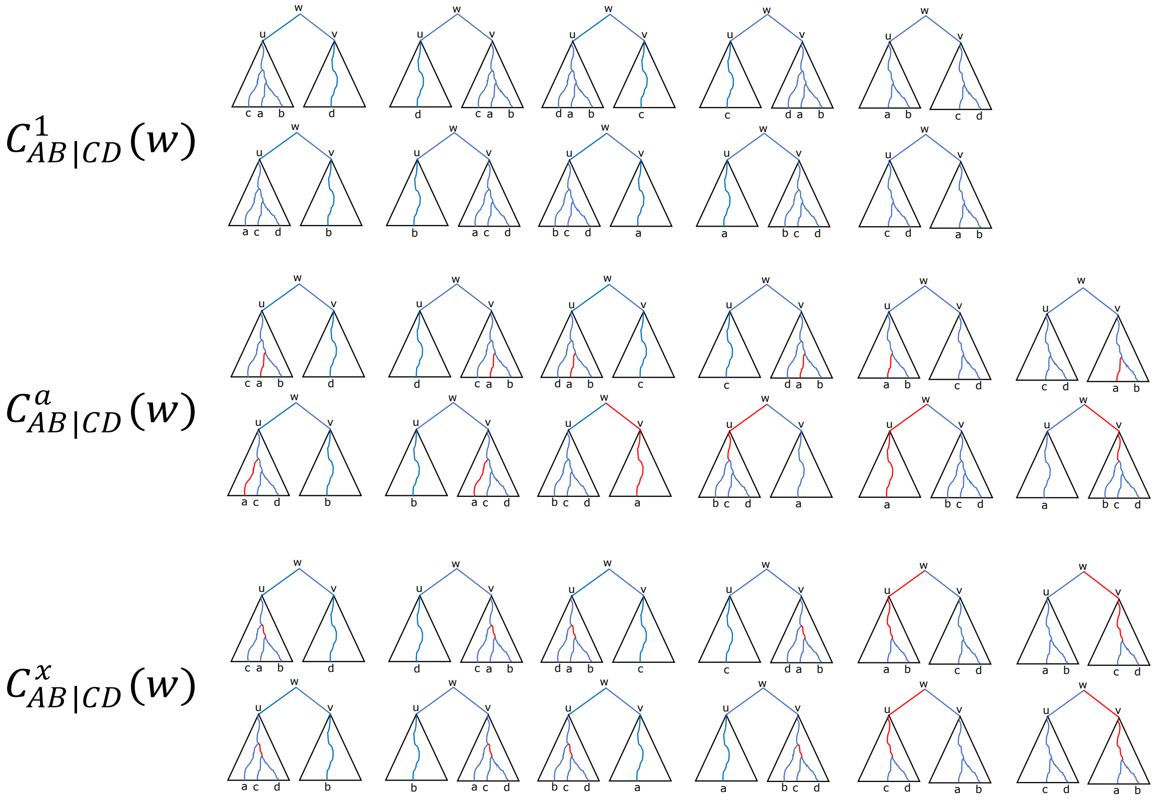

Figure S7: Illustration of recursive formulas (continued).

S3 Details of the Experimental Study

S3.1 Quartet Simulations

We used a modified version of SimPhy (Mallo et al., 2016), available at https://github.com/ytabatabaee/CASTLES/tree/main/simulation_files, that outputs species trees with substitution units branch lengths. We simulated quartet species trees and true gene trees under six different model conditions using the following commands:

```
q=no_variation # Homogeneous
./simphy -rs 200 -rl f:10000 -rg 1 -sb f:0.0001 -sd f:0 -st f:400 -sl f:4 -so f:0
-si f:1 -sp f:200 -su ln:-9,0.5 -hs f:10000 -hl f:10000 -hh f:10000 -hg f:10000
-cs 12964 -v 4 -o $q -ot 0 -op 1 -od 1 > log-$q.txt

q=only_hs # Sp
./simphy -rs 200 -rl f:10000 -rg 1 -sb f:0.0001 -sd f:0 -st f:400 -sl f:4 -so f:0
-si f:1 -sp f:200 -su ln:-9,0.5 -hs ln:1.5,1 -hl f:10000 -hh f:10000 -hg f:10000
-cs 12964 -v 4 -o $q -ot 0 -op 1 -od 1 > log-$q.txt

q=only_hl # Loc
./simphy -rs 200 -rl f:10000 -rg 1 -sb f:0.0001 -sd f:0 -st f:400 -sl f:4 -so f:0
-si f:1 -sp f:200 -su ln:-9,0.5 -hs f:10000 -hl ln:1.5,1 -hh f:10000 -hg f:10000
-cs 12964 -v 4 -o $q -ot 0 -op 1 -od 1 > log-$q.txt

q=hs_hl # Sp,Loc
./simphy -rs 200 -rl f:10000 -rg 1 -sb f:0.0001 -sd f:0 -st f:400 -sl f:4 -so f:0
-si f:1 -sp f:200 -su ln:-9,0.5 -hs ln:1.5,1 -hl ln:1.5,1 -hh f:10000 -hg f:10000
-cs 12964 -v 4 -o $q -ot 0 -op 1 -od 1 > log-$q.txt

q=hs_hl_hg # Sp,Loc,Sp/Loc
./simphy -rs 200 -rl f:10000 -rg 1 -sb f:0.0001 -sd f:0 -st f:400 -sl f:4 -so f:0
-si f:1 -sp f:200 -su ln:-9,0.5 -hs ln:1.5,1 -hl ln:1.5,1 -hh f:10000 -hg ln:1.5,1
-cs 12964 -v 4 -o $q -ot 0 -op 1 -od 1 > log-$q.txt

q=hs_hl_hg_highr # Sp,Loc,Sp/Loc,highILS
./simphy -rs 200 -rl f:10000 -rg 1 -sb f:0.0001 -sd f:0 -st f:400 -sl f:4 -so f:0
-si f:1 -sp f:800 -su ln:-9,0.5 -hs ln:1.5,1 -hl ln:1.5,1 -hh f:10000 -hg ln:1.5,1
-cs 12964 -v 4 -o $q -ot 0 -op 1 -od 1 > log-$q.txt
```

Table S5: Parameters used in SimPhy quartet simulations for all model conditions.

Arg.	Description	Value
RS	Number of replicates	200
RL	Number of loci	10000
RG	Number of genes	1
ST	Maximum tree length	400
SL	Number of taxa	4
SB	Speciation rate	0.0001
SD	Extinction rate	0
SP	Global population size	200 or 800
SU	Global substitution rate	LogNormal (-9,0.5)
HS	Species-specific branch rate heterogeneity modifiers	1 or LogNormal (1.5,1)
HL	Gene-family-specific rate heterogeneity modifiers	1 or LogNormal (1.5,1)
HG	Gene by lineage specific rate heterogeneity modifiers	1 or LogNormal (1.5,1)
HH	Gene-by-lineage-specific locus tree parameter	1
SO	Outgroup branch length relative to half the tree length	0 (no outgroup)
CS	Random number generator seed	12964

Table S6: Characteristics of the quartet simulation model conditions. AD stands for average discordance (RF distance) between the model species tree and true gene trees.

Condition	SP	HS	HL	HG	AD
Homogeneous	200	1	1	1	0.28
Sp	200	LogNormal (1.5,1)	1	1	0.30
Loc	200	1	LogNormal (1.5,1)	1	0.27
Sp,Loc	200	LogNormal (1.5,1)	LogNormal (1.5,1)	1	0.29
Sp,Loc,Sp/Loc	200	LogNormal (1.5,1)	LogNormal (1.5,1)	LogNormal (1.5,1)	0.29
Sp,Loc,Sp/Loc,highILS	800	LogNormal (1.5,1)	LogNormal (1.5,1)	LogNormal (1.5,1)	0.51

S3.2 Software Commands and Version Numbers

In this section, we bring the details of the experimental pipeline and software commands. The code for CASTLES as well as the scripts for error and distance matrix calculation use functions from DendroPy (Sukumaran and Holder, 2010). All experiments were run on the University of Illinois campus cluster.

S3.2.1 Branch Length Estimation

We used the following commands to estimate branch lengths in substitution units on a given species tree topology:

- **CASTLES**: Running CASTLES is a two-step approach: 1) Annotate branches of the species tree with mean quartet branch lengths around it using the tool ASTER, and 2) assign final branch lengths to each branch using the `castles.py` (v1.0.0) code available at github.com/ytabatabaee/CASTLES/blob/main/castles.py.

  Step 1) The algorithm to annotate branches with mean lengths of quartets around it is implemented in ASTER (v1.13.2.4) available at https://github.com/chaoszhang/ASTER. To annotate a fixed species tree topology with quartet statistics for each branch, we ran it with the option `-C` to score a fixed species tree specified after `-c`. Note that ASTER needs to be complied with a particular option for this annotation to work:

  ```
  g++ -std=gnu++11 -D"ASTRALIV" -march=native -Ofast -pthread
      src/astral.cpp -o bin/astral
  ```

  Assuming this version is used, we used the following command, where the annotated tree is printed to the log file:

  ```
  astral -C -i <gene_tree> -c <species_tree> -o <output_path> > annotated.tre
  ```

  Particularly, when we have multiple individuals per species and the individual names do not match the species names, we run the following command:

  ```
  astral -C -i <gene_tree> -m <name_map>
      -c <species_tree> -o <output_path> > annotated.tre
  ```

  where the "name map" file contains maps from individual names to species names in the following format:

  ```
  individual_name1    species_name1
  individual_name2    species_name2
  individual_name3    species_name3
  ...
  ```

  Step 2) The annotated tree is given to CASTLES to compute branch lengths in substitution units from these quartet statistics. To run CASTLES, we used the following command (note that the log file from ASTER is directly passed to CASTLES as input):

  ```
  python3 castles.py -t annotated.tre -g <gene_tree_path> -o <output_path>
  ```

- **FastME+Mean or FastME+Min**: Running FastME is also a two-step approach: 1) estimating the distance matrix, 2) inferring branch lengths.

Step 1) We used our custom script to compute patristic (path-length) distance matrices from gene trees in Phylip format. The core of this script is the `PhylogeneticDistanceMatrix` class from Dendropy. The script is available at https://github.com/ytabatabaee/CASTLES/blob/main/scripts/patristic_dist_matrix.py. The option `-m` specifies the type of distance matrix: `'avg'` and `'min'` compute a single squared matrix corresponding to the average and minimum path-length distances between pairs of nodes in a set of gene trees, respectively.

Step 2) We used FastME (v2.1.6.2) (Lefort et al., 2015) available at http://www.atgc-montpellier.fr/fastme/ to assign branch lengths with balanced minimum evolution (BME) criteria (specified with `-w BalLS`) to a given species tree topology, specified with `-u`, given a single distance matrix corresponding to average or minimum patristic distances computed across a set of gene trees, using the following command:

```
fastme-2.1.6.2-linux64 -i <dist_mat.phylip> -w BalLS -u <species_tree_path>
-o <output_path>
```

- **ERaBLE**: Similar to FastME, ERaBLE requires pre-computation of distances. We used the same script as FastME but with option `'all'` to compute one patristic distance matrix *per* gene, for a set of gene trees. We used the following command:

```
python3 patristic_dist_matrix.py -t <species_tree_path> -g <gene_tree_path>
-o <output_path> -m all
```

We then used ERaBLE (v1.0) (Binet et al., 2016) available at http://www.atgc-montpellier.fr/erable/. The input to ERaBLE is an unrooted tree topology in newick format, specified with the option `-t`, and a set of k distance matrices in Phylip format, each corresponding to a single gene tree, generated above. We used the following command:

```
erable -i  <dist_mat.phylip> -t <species_tree_path>  -o <output_path>
```

- **RAxML**: We used RAxML (v8.2.12) (Stamatakis, 2014) to estimate branch lengths on a given species tree topology, using a concatenated sequence alignment, with the option `-f e`. RAxML is available at https://github.com/stamatak/standard-RAxML. We used the following command:

```
raxmlHPC-PTHREADS -f e -t <species_tree_path> -m GTRGAMMA -s <alignment_path>
-n RES  -p 4321 -T 16
```

S3.2.2 Error Calculation

We used the script available at https://github.com/ytabatabaee/CASTLES/blob/main/scripts/ compare_trees_bl.py to compare branch lengths on two trees, and compute the root mean squared error (RMSE) and the average logarithmic error between the trees for all branches. We used the following command:

```
python3 compare_trees_bl.py -t1 <true_species_tree_path> -t2 <est_species_tree_path>
```

which tabulates the set of true and estimated branch lengths. These data are then analyzed using an R script available at https://github.com/ytabatabaee/CASTLES/blob/main/results/draw.R Note that for all methods, before computing the error, negative and zero branch lengths are replaced with 1e-6.

The formulas for the error metrics used in this study for a species tree $\mathcal{T}$ with b branches are as follows, where t_i and $\hat{t_i}$ are the true and estimated lengths of branch i in SU respectively:

- Bias: $\frac{1}{b}\sum_{i=1}^{b}(t_i - \hat{t_i})$
- Mean absolute error: $\frac{1}{b}\sum_{i=1}^{b}|t_i - \hat{t_i}|$
- Logarithmic error: $\frac{1}{b}\sum_{i=1}^{b}|log_{10}(t_i) - log_{10}(\hat{t_i})|$
- Root mean square error (RMSE): $\sqrt{\frac{1}{b}\sum_{i=1}^{b}(t_i - \hat{t_i})^2}$

S3.2.3 Runtime and Memory

We measure runtime as the total running time of all steps of each method, assuming that the estimated gene trees, alignments, and species tree topology are already available. This includes the time needed to calculate the distance matrices for ERaBLE and FastME, as well as the time needed for annotating trees with ASTER that is given as input to CASTLES. To measure the runtime and peak memory usage of a command `<cmd>`, we use the following command:

```
/usr/bin/time -v -o out.stat -f "\t%e\t%M" <cmd>
```

We record runtime from the elapsed wall clock time, and peak memory usage from maximum resident set size from the generated `out.stat` file.

S3.3 Biological Data Analysis

We analyzed the mammalian biological dataset of Song et al. (2012), which had 37 species (36 ingroups and one outgroup). The original dataset had 447 genes, but we used a processed version of the dataset with 424 genes available here, that had 23 mislabeled or outlier genes removed. We estimated an unrooted species tree using ASTRAL (v5.7.8) (Zhang et al., 2018) available at github.com/smirarab/ASTRAL using the following command.

```
java -jar astral.5.7.8.jar -i <gene-trees.tre> -o <species-tree.tre>
```

We then removed the outgroup (Chicken, specified with the name "GAL" in the dataset) from the ASTRAL tree, as our simulations show that branch length estimation methods benefit from the removal of outgroup, and then estimated branch lengths on the tree. All files associated with this analysis are available at https://github.com/ytabatabaee/CASTLES/tree/main/results/mammalian-biological-analysis.

S4 Additional Figures

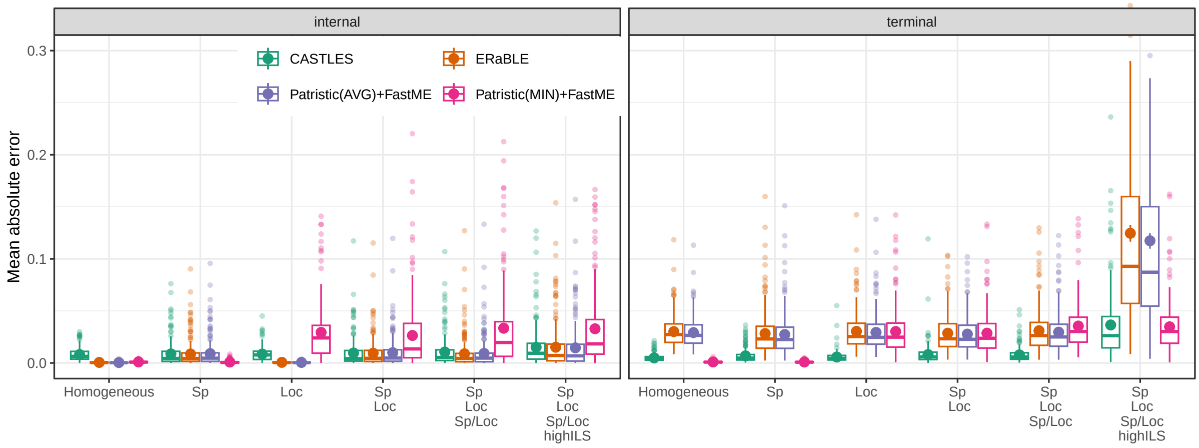

Figure S8: Mean absolute error on terminal and internal branches for simulated quartet datasets. The plot shows mean and standard error across 200 replicates, in addition to boxplots. The y-axes is cut at 0.3, eliminating a couple of outlier cases with unusually high errors (none from CASTLES). Figure 2.a in the main text shows the same error aggregated for terminal and internal branches.

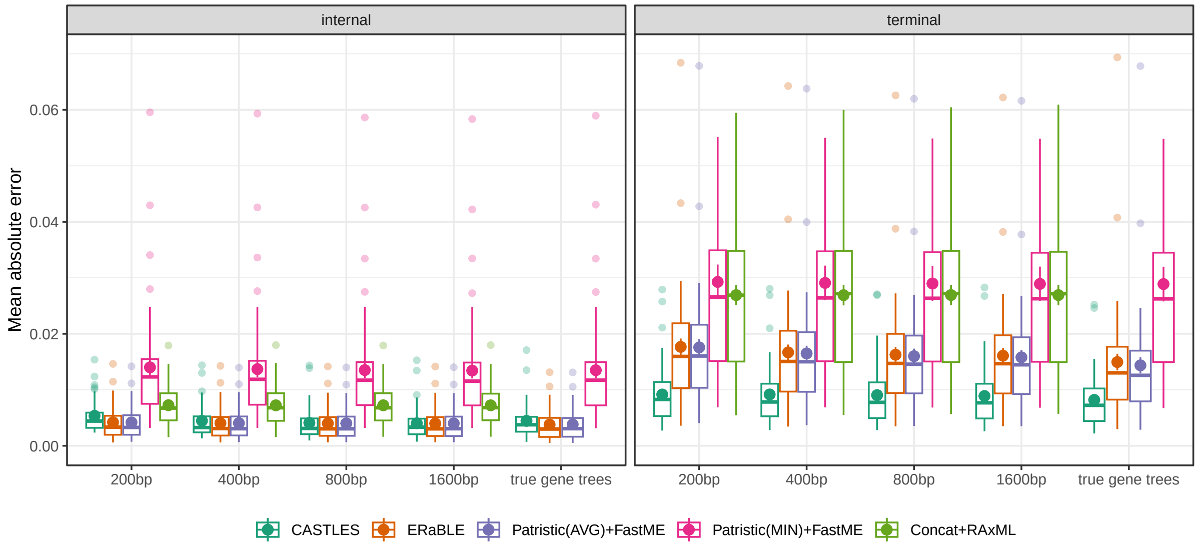

Figure S9: Mean absolute error on terminal and internal branches for simulated 101-taxon datasets. The plot shows mean and standard error across 50 replicates, in addition to boxplots. The y-axes is cut at 0.07, eliminating a few outlier cases with unusually high errors (none from CASTLES). Figure 3.a in the main text shows the same error aggregated for terminal and internal branches.

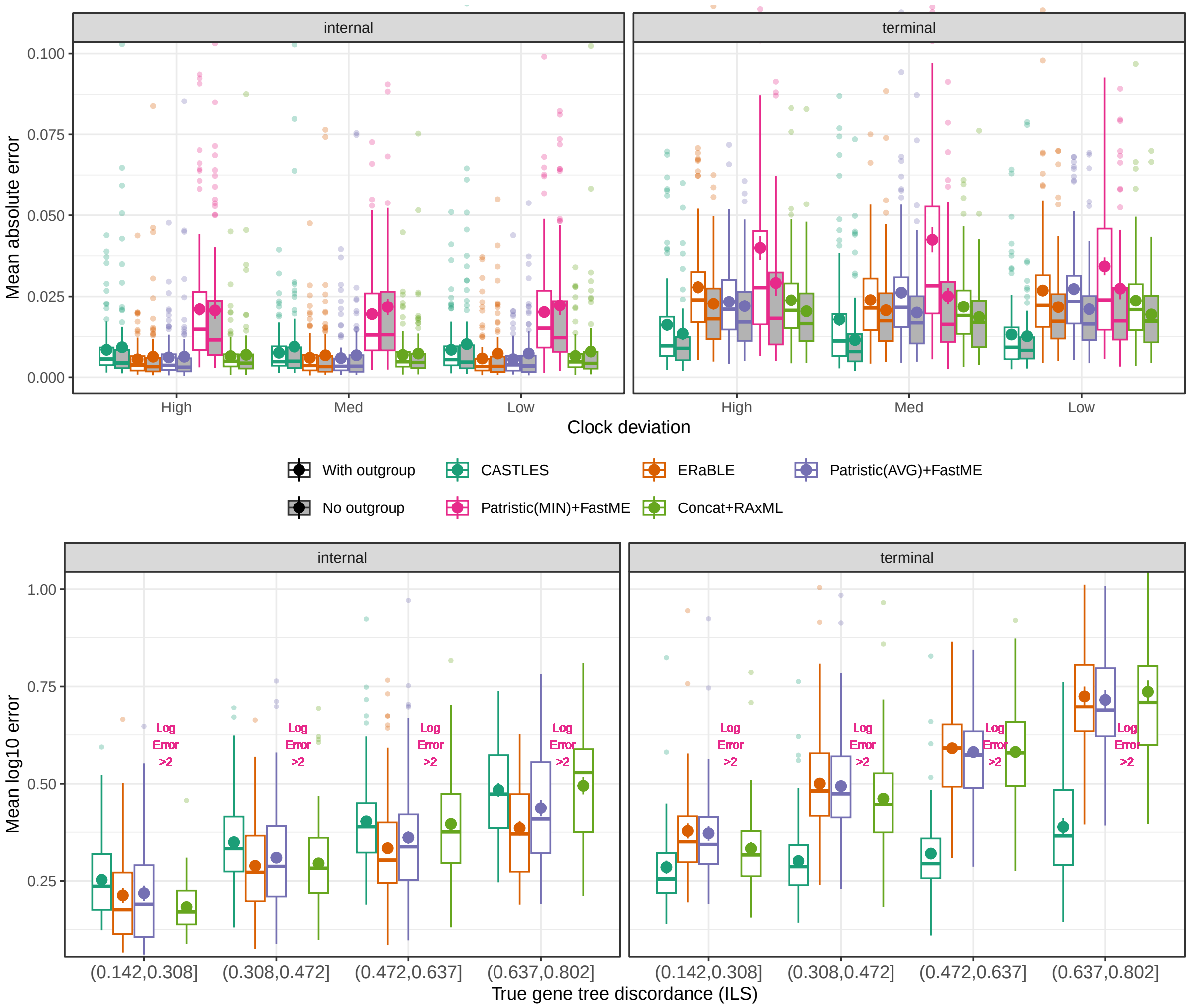

Figure S10: Mean absolute error (top) and mean log error (bottom) on terminal and internal branches for simulated MVRoot datasets. The plot shows mean and standard error across 100 replicates, in addition to boxplots. The y-axis is cut at 0.11, leaving a few outliers out of the graph (one from CASTLES). Patristic(MIN)+FastME has mean log error above 2 (see Fig. S18) and is excluded. Figure 4 in the main text shows the same errors aggregated for terminal and internal branches.

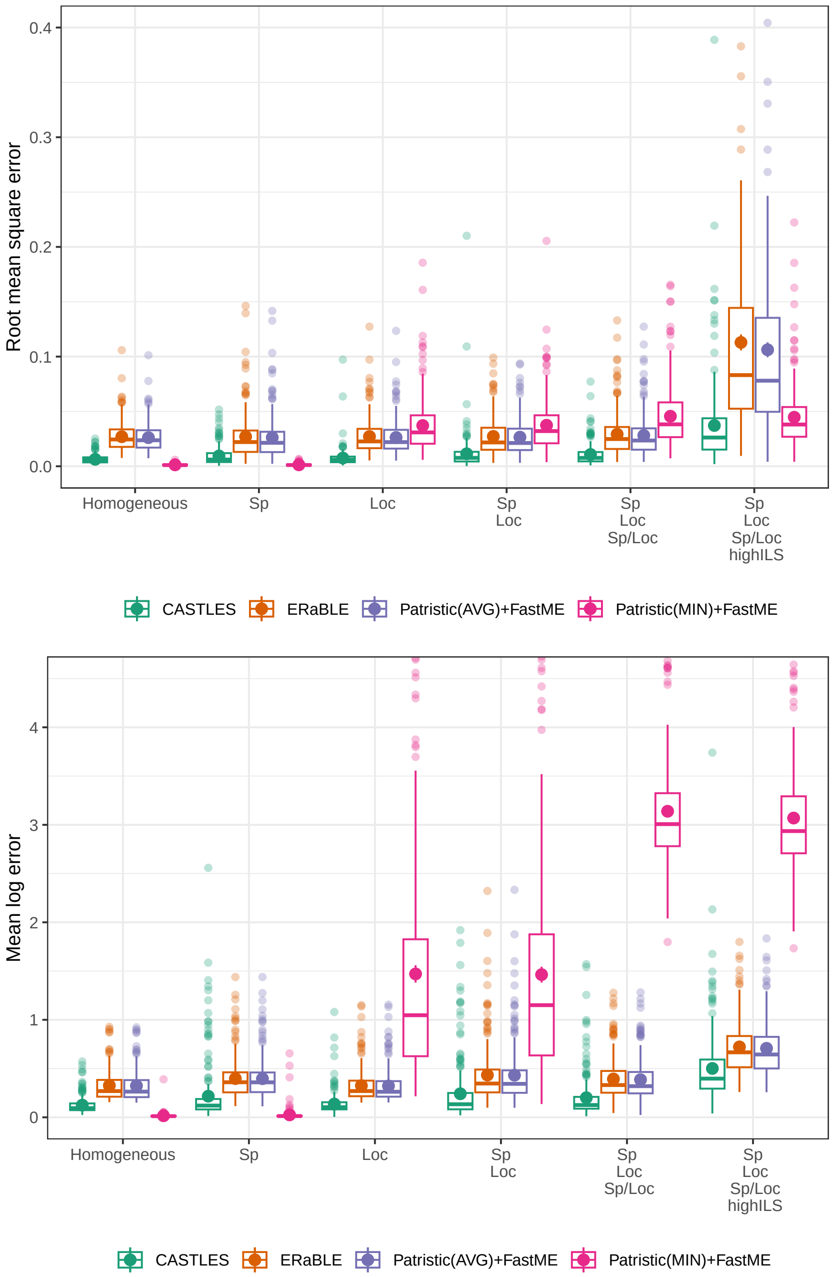

Figure S11: RMSE (top) and mean log error (bottom) of branch lengths estimated using different methods on simulated quartet datasets. Both panels show mean and standard deviation across 200 replicates, in addition to boxplots. The y-axes are cut at 0.4 and 4.5 for the top and bottom panels respectively, eliminating a few outlier cases with unusually high errors (none from CASTLES).

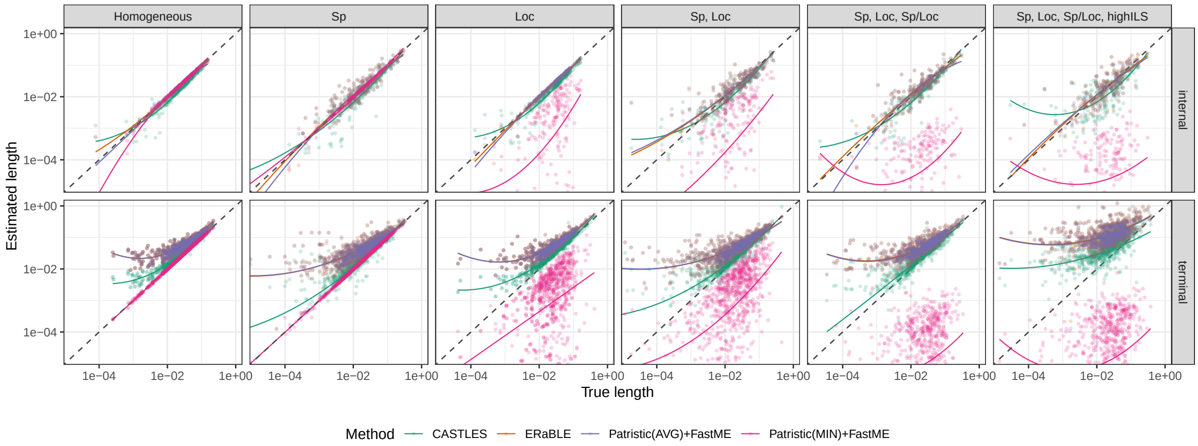

Figure S12: Correlations between true and estimated branch lengths on the quartet datasets. Each dot corresponds to a single branch in an unrooted quartet species tree, and the results are shown across 200 replicates (therefore 5×200 points for each condition and method). The lines show a fitted degree-two polynomial with smoothing.

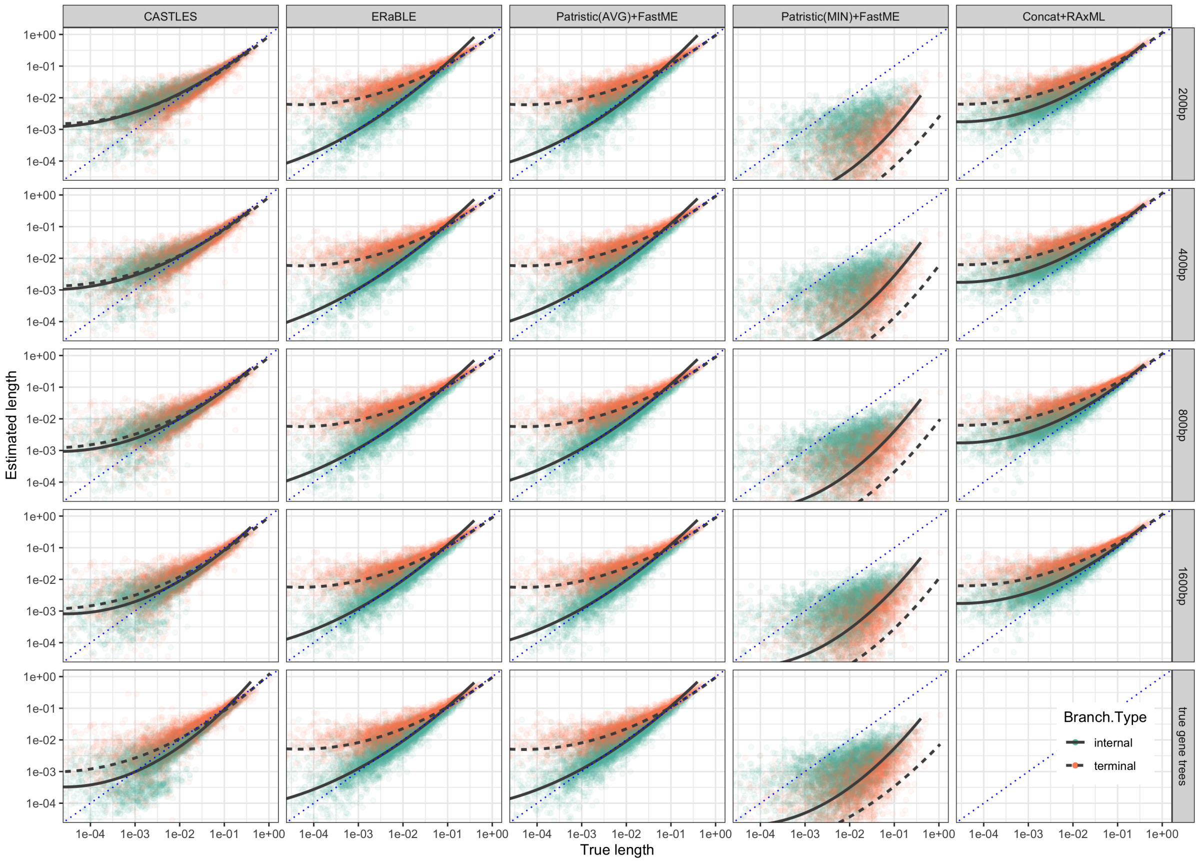

Figure S13: Correlations between true and estimated branch lengths on the S100 datasets. Each dot corresponds to a single branch in an unrooted 101-taxon species tree, and the results are shown across 50 replicates (therefore 50×199 points in each subfigure). The lines show a fitted degree-two polynomial with smoothing.

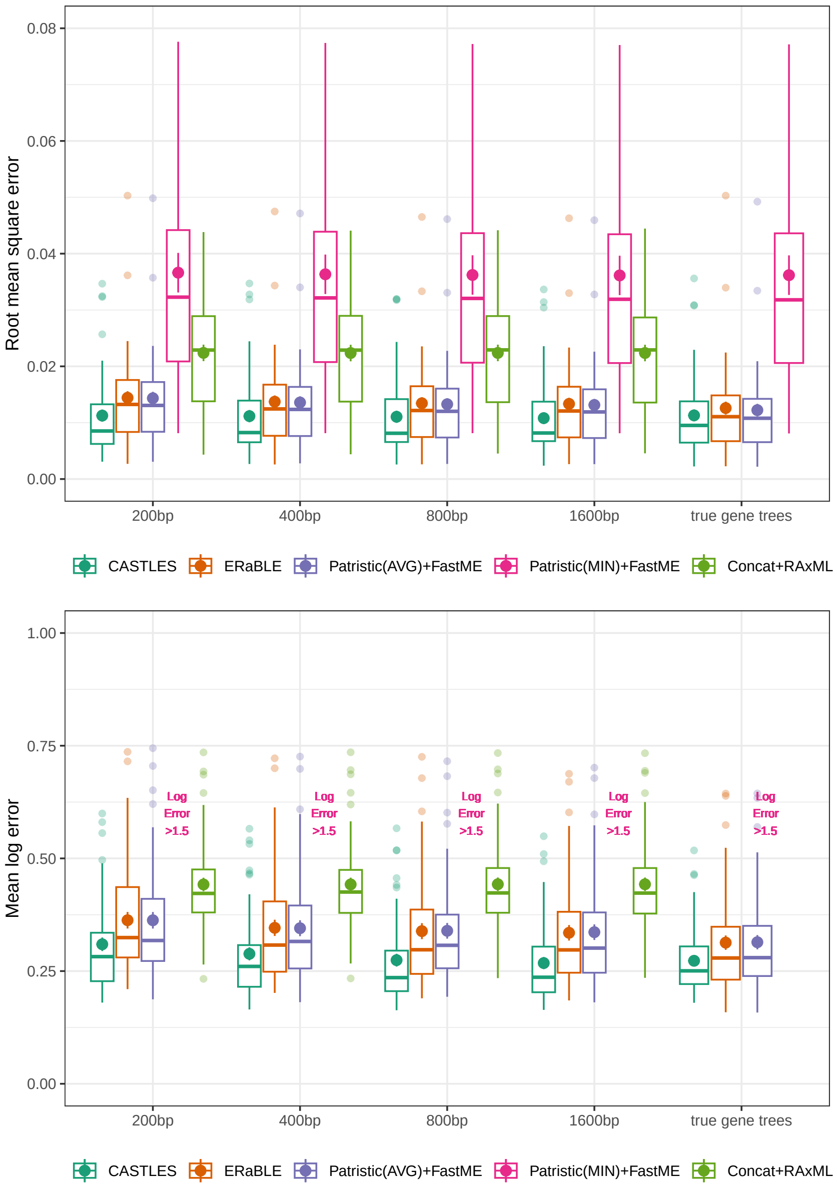

Figure S14: RMSE (top) and mean log error (bottom) of branch lengths estimated using different methods on simulated S100 datasets. Both panels show mean and standard deviation across 50 replicates, in addition to boxplots. The average GTEE level varies between zero (for true gene trees) to 23% (for 1600bp) and then to 55% (for the 200bp sequences). The average ILS level is 0.46 AD, and the number of genes is 1000.

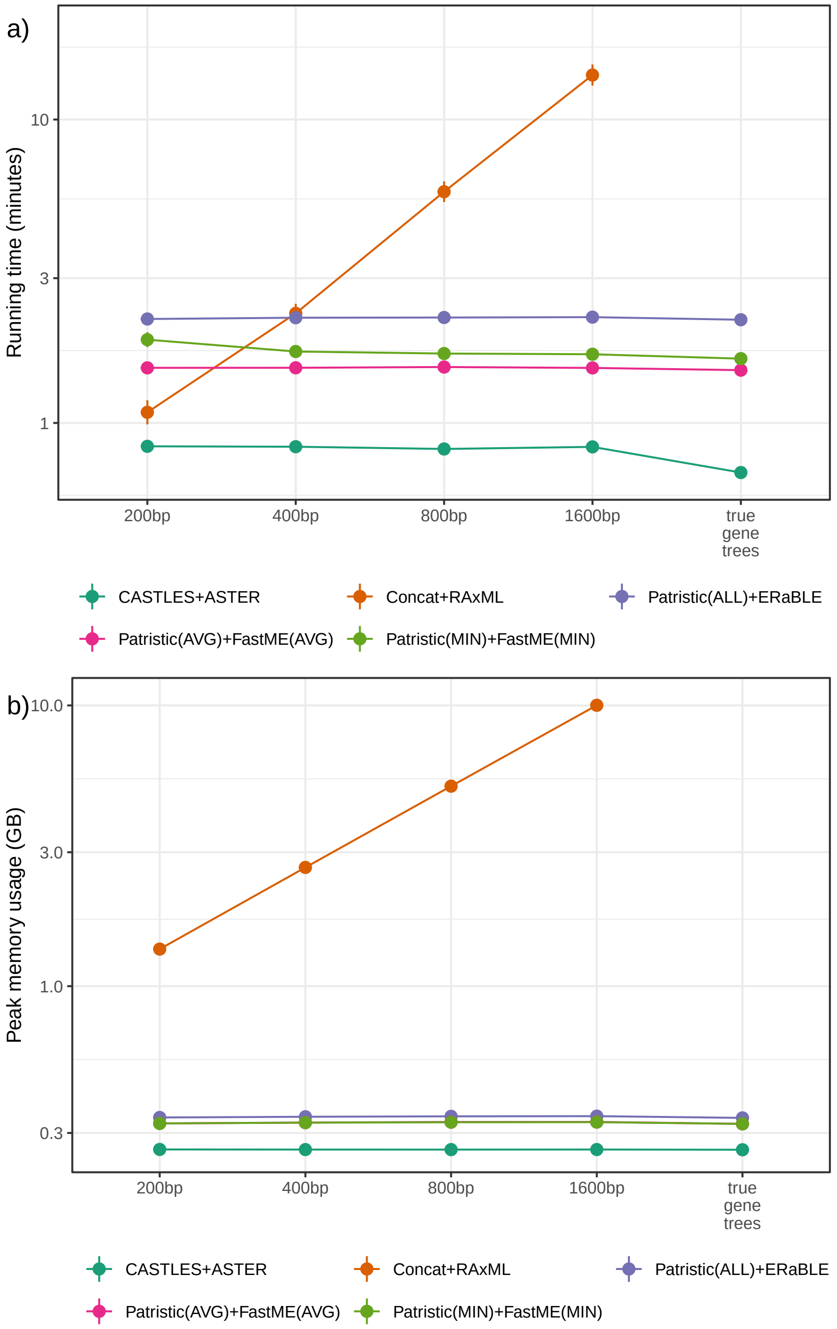

Figure S15: (a) Average total running time in minutes and (b) peak memory usage in gigabytes (both in log scale) of different branch length estimation pipelines on the 101-taxon datasets with 1000 genes. The runtimes are reported as the total running time of all steps for each method (see Sec. S3.2.3). The results are averaged over 50 replicates in each model condition.

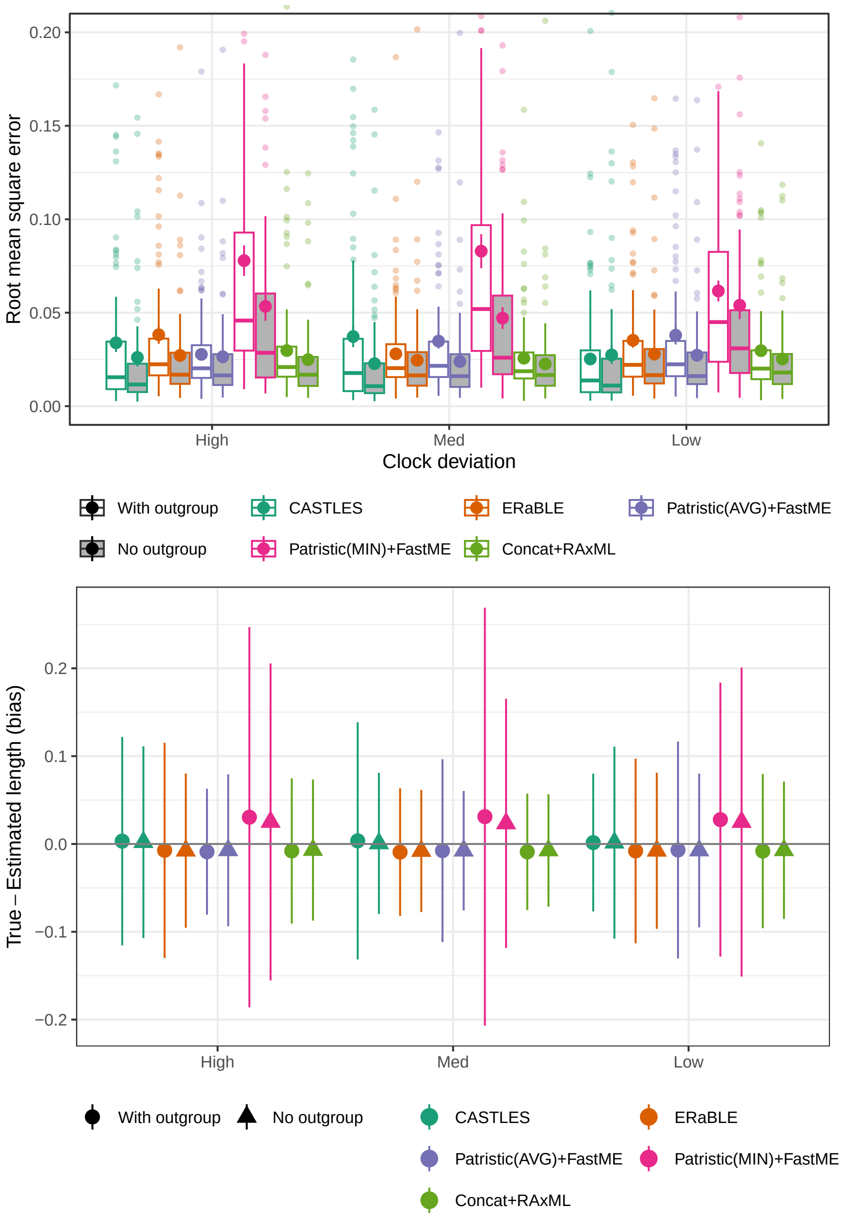

Figure S16: RMSE (top) and bias (bottom) of branch lengths estimated using different methods on MVRoot datasets. The top panel shows mean and standard deviation across 100 replicates, in addition to boxplots, and the bottom panel shows mean and standard deviation. The number of genes is 500 and the results are shown across 100 replicates.

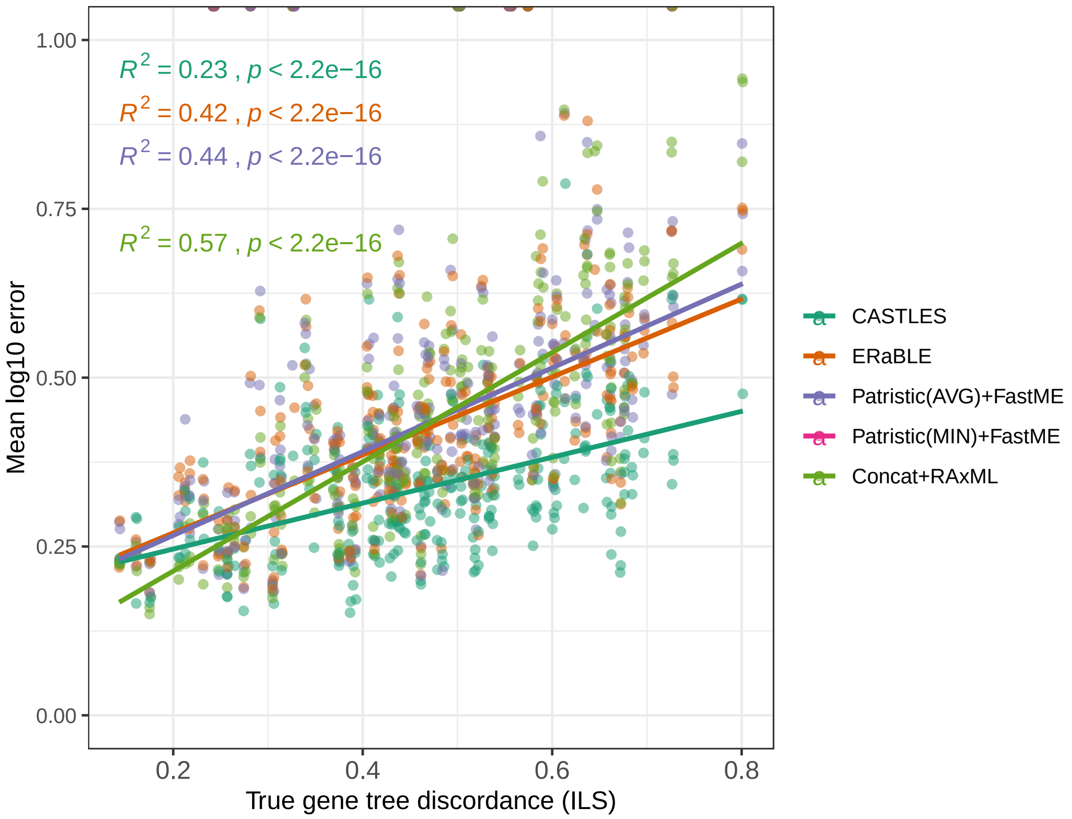

Figure S17: Mean log error (in base 10) of branch lengths estimated using different methods (excluding Patristic(MIN)+FastME) versus ILS measured using AD (mean RF distance of true gene trees to the model species tree) on the MVRoot dataset in the no-outgroup model conditions. The number of genes is 500 and the results are shown across 100 replicates.

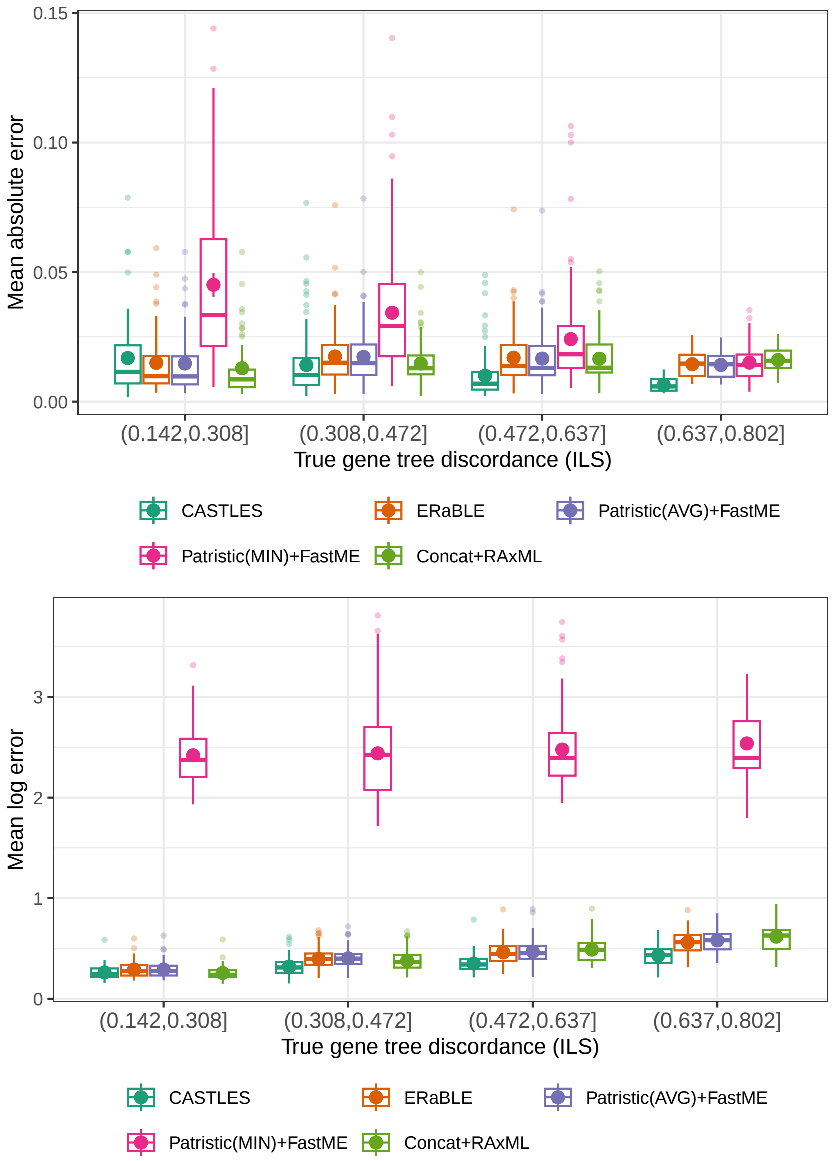

Figure S18: Mean absolute error (top) and mean log error (bottom) of branch lengths estimated using different methods on MVRoot datasets. Focusing on cases without outgroups, we divide replicates based on their level of true gene tree discordance due to ILS into four groups. The number of genes is 500 and the results are shown across 100 replicates. The main paper excluded Patristic(MIN)+FastME due to very high error.

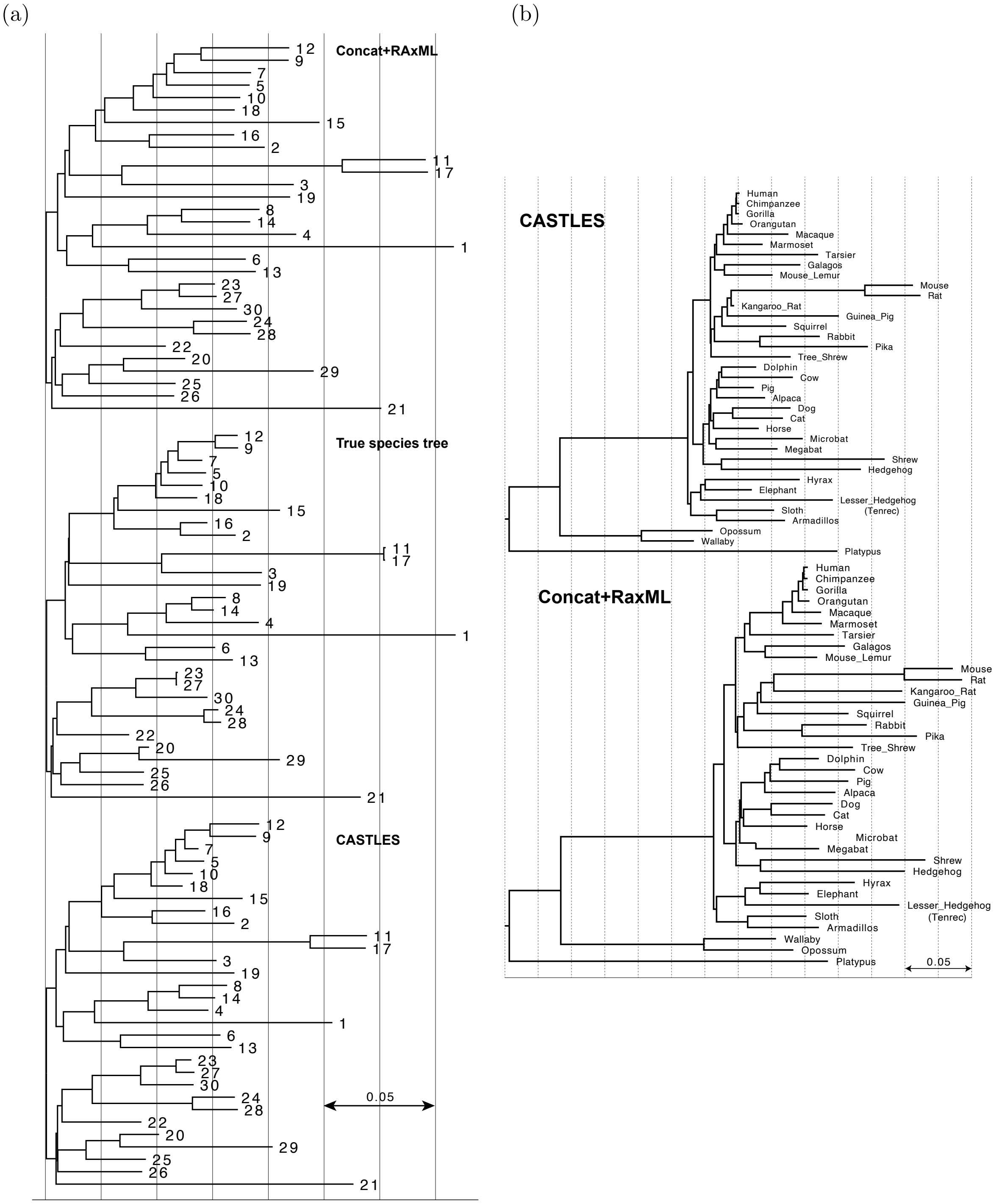

Figure S19: (a) Visualization of the true species tree (middle), and trees with branch lengths produced by CASTLES (bottom) and Concat+RAxML (top) for replicate 1 of MVRoot dataset in the default model condition (no outgroup, medium deviation from the strict clock). The ILS level and GTEE level for this replicate are 0.53 and 0.34 respectively. (b) Same methods applied on the real mammalian dataset (Song et al., 2012). Here, the outgroup (Chicken) is removed *before* drawing branch lengths on the tree. The trees are visualized using FigTree (Rambaut, 2023).

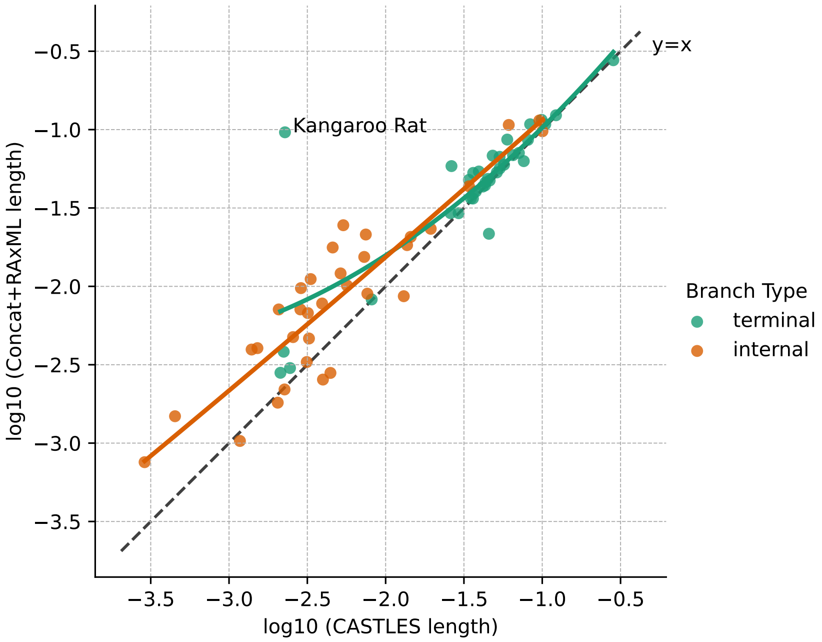

Figure S20: Correlations between branch lengths produced by CASTLES and Concat+RAxML on the 37-taxon mammalian dataset (Song et al., 2012). The branch lengths are drawn on a species tree topology constructed by ASTRAL. The outgroup (Chicken) is removed before drawing branch lengths on the tree. The number of genes used in the analysis is 424. Each point corresponds to a single branch in the species tree. The colored lines show a fitted degree-two polynomial.

References

Allman, E. S., Degnan, J. H., and Rhodes, J. A. (2011). Identifying the rooted species tree from the distribution of unrooted gene trees under the coalescent. *Journal of mathematical biology*, 62:833–862.

Binet, M., Gascuel, O., Scornavacca, C., P. Douzery, E. J., and Pardi, F. (2016). Fast and accurate branch lengths estimation for phylogenomic trees. *BMC Bioinformatics*, 17(1):23.

Kingman, J. F. (1982). On the genealogy of large populations. *Journal of applied probability*, 19(A):27–43.

Lefort, V., Desper, R., and Gascuel, O. (2015). FastME 2.0: a comprehensive, accurate, and fast distance-based phylogeny inference program. *Molecular biology and evolution*, 32(10):2798–2800.

Mallo, D., De Oliveira Martins, L., and Posada, D. (2016). SimPhy : Phylogenomic Simulation of Gene, Locus, and Species Trees. *Systematic Biology*, 65(2):334–344.

Rambaut, A. (2023). FigTree (v1.4.4). http://tree.bio.ed.ac.uk/software/figtree/, Date last accessed: January 19, 2023.

Rosenberg, N. A. (2002). The probability of topological concordance of gene trees and species trees. *Theoretical Population Biology*, 61(2):225–247.

Sayyari, E. and Mirarab, S. (2016). Fast coalescent-based computation of local branch support from quartet frequencies. *Molecular biology and evolution*, 33(7):1654–1668.

Song, S., Liu, L., Edwards, S. V., and Wu, S. (2012). Resolving conflict in eutherian mammal phylogeny using phylogenomics and the multispecies coalescent model. *Proceedings of the National Academy of Sciences*, 109(37):14942–7.

Stamatakis, A. (2014). RAxML version 8: a tool for phylogenetic analysis and post-analysis of large phylogenies. *Bioinformatics*, 30(9):1312–1313.

Sukumaran, J. and Holder, M. T. (2010). DendroPy: a Python library for phylogenetic computing. *Bioinformatics*, 26(12):1569–1571.

Tavaré, S. (1984). Line-of-descent and genealogical processes, and their applications in population genetics models. *Theoretical Population Biology*, 26(2):119–164.

Zhang, C., Rabiee, M., Sayyari, E., and Mirarab, S. (2018). ASTRAL-III: polynomial time species tree reconstruction from partially resolved gene trees. *BMC Bioinformatics*, 19(S6):153.
